# Supercarriers of antibiotic resistome in a world’s large river

**DOI:** 10.1186/s40168-022-01294-z

**Published:** 2022-07-28

**Authors:** Jiawen Wang, Rui Pan, Peiyan Dong, Shufeng Liu, Qian Chen, Alistair G. L. Borthwick, Liyu Sun, Nan Xu, Jinren Ni

**Affiliations:** 1 College of Environmental Sciences and Engineering, Peking University; Key Laboratory of Water and Sediment Sciences, Ministry of Education, Beijing, 100871 People’s Republic of China; 2 State Environmental Protection Key Laboratory of All Material Fluxes in River Ecosystems, Beijing, 100871 People’s Republic of China; 3 State Key Laboratory of Plateau Ecology and Agriculture, Qinghai University, Xining, 810016 People’s Republic of China; 4 Institute of Infrastructure and Environment, School of Engineering, The University of Edinburgh, The King’s Buildings, Edinburgh, EH9 3JL UK; 5 School of Engineering, Computing and Mathematics, University of Plymouth, Drake Circus, Plymouth, PL4 8AA UK; 6 School of Environment and Energy, Peking University Shenzhen Graduate School, Shenzhen, 518055 People’s Republic of China

**Keywords:** Antibiotic resistance genes, Host, Human pathogen bacteria, Yangtze River

## Abstract

**Background:**

Antibiotic resistome has been found to strongly interact with the core microbiota in the human gut, yet little is known about how antibiotic resistance genes (ARGs) correlate with certain microbes in large rivers that are regarded as “terrestrial gut.”

**Results:**

By creating the integral pattern for ARGs and antibiotic-resistant microbes in water and sediment along a 4300-km continuum of the Yangtze River, we found that human pathogen bacteria (HPB) share 13.4% and 5.9% of the ARG hosts in water and sediment but contribute 64% and 46% to the total number of planktonic and sedimentary ARGs, respectively. Moreover, the planktonic HPB harbored 79 ARG combinations that are dominated by “natural” supercarriers (e.g., *Rheinheimera texasensis* and *Noviherbaspirillum* sp. Root189) in river basins.

**Conclusions:**

We confirmed that terrestrial HPB are the major ARG hosts in the river, rather than conventional supercarriers (e.g., *Enterococcus* spp. and other fecal indicator bacteria) that prevail in the human gut. The discovery of HPB as natural supercarriers in a world’s large river not only interprets the inconsistency between the spatial dissimilarities in ARGs and their hosts, but also highlights the top priority of controlling terrestrial HPB in the future ARG-related risk management of riverine ecosystems globally.

Video Abstract

**Supplementary Information:**

The online version contains supplementary material available at 10.1186/s40168-022-01294-z.

## Background

The dissemination of antibiotic resistance genes (ARGs) and their hosts has been accelerated by extensive overuse of antibiotics around the world [1]. Antibiotic-resistant microorganisms (ARMs) that carried single/multiple ARGs can infect humans by contacting or entering the food chain and possibly trigger public health risks of severe infection and high mortality [2, 3]. In the past decades, great efforts have been made to understand the interactions between ARGs and microorganisms in the human gut [4–6]. More recently, explorations have been extended to other anthropogenic systems such as agricultural fertilized soils [7], wastewater treatment plants (WWTPs) [8], and even complex natural systems like reservoirs [9], lakes [10], and rivers [11].

In the human gut, ARGs were found mainly conferring resistance to tetracycline, multidrug, and macrolide-lincosamide-streptogramin, mostly hosted by *Escherichia coli*, *Streptococcus salivarius*, and *Bacteroides vulgatus* [4–6]. Among the antibiotic-resistant hosts, human pathogenic bacteria (HPB) such as *Escherichia*, *Helicobacter*, *Neisseria*, and *Klebsiella* were highly noted due to their potential to induce multiple human diseases [12]. However, ARGs would demonstrate resistance to diverse antibiotics in varying systems beyond human/animal guts, potentially being hosted by different kinds of ARMs and HPB. In soil systems, for example, ARGs exhibit resistance to beta-lactams, aminoglycosides, amphenicols, sulfonamides, and tetracyclines [3], owing to the long-term application of manure fertilizer containing antibiotic residues. Consequently, HPB such as *Salmonella*, *Bacteroidales*, *Campylobacter*, *Shigella*, and *Enterococcus* could become supercarriers in different soil systems [13, 14]. WWTPs provide another example by acting as typical anthropogenic hotspots with the enrichment of multiple ARGs resistant to sulfonamide, beta-lactam, and tetracycline, facilitating horizontal gene transfer of ARGs among environmental bacteria and human pathogens and serving as an important pathway to transport antibiotic resistance into aquatic ecosystems [8, 15].

In natural rivers (analogous to a “terrestrial gut”) connecting continents and the oceans, ARGs could be from sewage discharge and soils via surface runoffs or be disseminated to various surrounding environments and human individuals [16]. Previous studies have mostly focused on the occurrence, composition, and distribution of riverine ARGs and their potential hosts in relatively small streams [17], tributaries [18], river reaches [11], and estuarine areas [19]. In a few studies [11, 19], certain riverine ARGs and hosts have been identified, depending on various environmental factors and human activities. Even so, the present understanding of the relationship between ARGs and core microbiota in riverine systems is far from satisfactory.

To address the above knowledge gap, we firstly provide an integral biogeographic pattern for both ARGs and their hosts along the Yangtze River, the third largest river in the world (Fig. S1). Intriguingly, our study reveals that terrestrial HPB were the supercarriers of antibiotic resistome in the Yangtze, which are essentially different from conventional HPB prevalent in the human gut. We confirm that riverine HPB contribute 46~64% to the total ARGs through harboring 79 ARG combinations in spite of their minor presence in ARG hosts (5.9~13.4%). These findings are of the utmost significance in mitigating health risks associated with antibiotic-resistant bacteria in global large river ecosystems.

## Methods

### Sample collection

The Yangtze River, the world’s third longest river, originates in the Qinghai-Tibet Plateau and flows eastwards into the East China Sea. The river has a total length of 6300 km and a drainage basin of 1.8 million km^2^, experiencing various changes in landform type and hydrological regime [20]. To investigate the integral complete biogeographic patterns of ARGs and their hosts throughout the whole river, paired water and sediment samples were synchronously (i.e., within 1 week) collected at 49 national hydrologic monitoring stations along the mainstream and major tributaries of the Yangtze in March (spring) and October (autumn) 2014, and no extreme weather event occurred before and during the sample collection. A synchronous sampling of water and sediment is laborious and rarely implemented in large river systems. At some sites with heterogeneous sediments, replicates are necessary though fewer parallel water samplings are acceptable under the restricted conditions with the steep terrain and rapid flow [21, 22]. In fact, the mixture of water samples taken over a cross-section at a specific site (according to national hydrologic sampling specification) is more representative in a large river like the Yangtze. As a result, 1~4 samples (considering the significance of replicates) for sediments were undertaken at many monitoring sites particularly where heterogeneous sediments were found. Meanwhile, replicates for water were also conducted at three monitoring sites with the extremely high velocity and turbulent flow of the Yangtze River, and other “single” water samples were collected as a mixture of multiple subsamples across the section at any specific monitoring site. Noting that some samples could not be taken at several monitoring sites due to their steep terrain and rapid flow conditions, a total of 219 samples comprising 87 water and 132 sediment samples were finally obtained for further analysis. Detailed information about these samples was provided in Additional file 2: Table S1.

At each monitoring site, 10 L of water was collected into sterile PET bottles using a plexiglass water sampler at a depth of approximately 0.5 m below the water surface. Then, the water samples were immediately transported to an adjacent laboratory. One part of the water samples from each monitoring site was stored at 0–4 °C for further physicochemical analysis, and the other part for DNA extraction was filtered through 0.22-μm polycarbonate membranes (Millipore, USA) within 24 h. The filtered membranes were kept frozen at − 80 °C until further DNA extraction. Meanwhile, paired sediment samples were collected from the top layer (0–5 cm) of the river bed and placed into 50-ml sterilized polypropylene tubes. Sediments for molecular analysis were placed in dry ice, immediately brought back to the laboratory, and stored at − 80 °C. Another 1 kg of sediments was packaged within polyethylene bags for physicochemical analysis.

### Physicochemical analysis

Physicochemical analysis was conducted for all water and sediment samples in triplicate [23]. For each water sample, pH, water temperature (WT), pH, dissolved oxygen (DO), suspended solids (SS), total nitrogen (TN), nitrate-nitrogen (NO_3_^−^-N), ammonium-nitrogen (NH_4_^+^-N), total phosphorus (TP), and dissolved organic carbon (DOC) were measured according to the Environmental Quality Standards for Surface Water (GB3838-2002) recommended by the Ministry of Ecology and Environment of China [24]. As for the sediment samples, pH, TN, NO_3_^−^-N, NH_4_^+^-N, TP, and total organic carbon (TOC) were also determined as described by our previous studies [21–23]. Meanwhile, a total of 26 antibiotics, covering 1 type of aminoglycoside, 1 bacitracin, 3 beta-lactams, 2 chloramphenicols, 3 macrolides, 4 quinolones, 8 sulfonamides, and 4 tetracyclines, were measured in water and sediment. The averaged concentrations of antibiotics were provided in Additional file 2: Table S2. In addition, elevation (E), longitude, and latitude for each sampling site were also recorded by a handheld GPS (Magellan, USA).

### DNA extraction and metagenomic sequencing

Environmental DNA was extracted from each sample (using mixed filtered membranes or 0.5 g sediments) multiple times with the FastDNA® SPIN Kit for Soil (MP Biomedicals, USA) following the manufacturer’s instructions. The DNA extracts were mixed together, and the quality and concentrations of DNA were evaluated using the NanoDrop ND-2000 instrument (Thermo Fisher Scientific, USA). The high-quality DNA (DNA amount > 1 μg and concentration > 10 ng μL^−1^) was used for library construction by utilizing the NEBNext Ultra DNA Library Prep Kit for Illumina (NEB, USA) and then submitted for sequencing on an Illumina Hiseq 4000 platform using the paired-end (2 × 150 bp) strategy (Majorbio Company, Shanghai, China). During the DNA extraction and sequencing processes, three negative controls were applied to monitor any potential contamination, and no quantifiable DNA was detected for further molecular analysis.

### Bioinformatics analysis

#### Metagenomic assembly

Raw metagenomic reads of each sample were quality-filtered, trimmed, and screened using Sickle (-q 20 -l 50) (https://github.com/najoshi/sickle) and NGS QC Toolkit (-l 70 -s 20) [25]. Then, high-quality clean reads were assembled into contigs using IDBA-UD with default parameters [26], and contigs longer than 500 bp were used for predicting open reading frames (ORFs) by MetaGeneMark [27]. Clean reads were annotated by comparison with the SILVA small subunit database (release 132) using BLASTN (-max_target_seqs 1 -e 10^-20^) [28]. The sequencing depth of each dataset was provided in Additional file 2: Table S3. The BLAST results were used to extract 16S rRNA gene-like sequences assigned to SILVA taxonomies by QIIME [29].

#### Identification of ARG-like sequences

All metagenomic clean reads were searched for ARGs against the DeepARG-DB database using DIAMOND with default probability values [30]. Identified ARG-like sequences were automatically categorized into 31 “ARG types” and 2195 “ARG subtypes” for further analysis. To investigate the ARG biogeography in the Yangtze River, the abundance of ARGs (copy of ARG per copy of 16S-rRNA gene) was normalized using the following equation [31]:

1 $$\mathrm{ARGs}\ \mathrm{abundance}=\sum_i^n\frac{N_{i\ \left(\mathrm{ARG}-\mathrm{like}\ \mathrm{sequence}\right)}\times \frac{L_{\mathrm{reads}}}{L_{\mathrm{i}\ \left(\mathrm{ARG}\ \mathrm{reference}\ \mathrm{sequence}\right)}}}{N_{16\mathrm{s}\ \mathrm{sequence}}\times \frac{L_{\mathrm{reads}}}{{\mathrm{L}}_{16\mathrm{S}\ \mathrm{sequence}}}}$$

where *N*_*i* (ARG-like sequence)_ is the number of ARG-like reads mapped to a specific ARG reference sequence, *L*_reads_ is the sequence length (bp) of the Illumina reads, *L*_*i* (ARG reference sequence)_ is the sequence length of the corresponding target ARG reference sequence, *N*_16S sequence_ is the number of the 16S rRNA gene sequence, and *L*_16S sequence_ is the average sequence length of 16S rRNA genes (1432 bp) in the Greengenes database [32].

#### Taxonomy annotation of ARG-carrying contigs

Predicted ORFs of each ARG-carrying contig (ACC) were searched against the DeepARG-DB database using DIAMOND (DeepARG-LS model, --sensitive -e 10^-10^ -f 6 -k 1). ARG-like ORFs were identified as those ORFs with the best hit in the DeepARG-DB database exceeding 80% identity over 70% of the query coverage [33]. Then, the ARG-like ORFs were compared against the local NCBI non-redundant database using DIAMOND (--sensitive -e 10^-10^ -f 6 -k 1), and the results were parsed using MEGAN6 [34]. For each ARG-carrying contig, the taxonomic assignment was achieved if more than 50% of the ARG-like ORFs within a contig were classified to the same species, then the ACC would be annotated to that species, and this ARG-carrying microbe was regarded as a potential ARG host [33]. Moreover, the ARG-carrying contigs were then searched against the previously established pathogen database [35] to identify potential human pathogenic bacteria (HPB) at the species level. Meanwhile, the ACCs were also compared against the virulence factor database to identify virulence factor genes (VFGs) [36], and those ARG hosts carrying any VFGs were also classified as HPB. To assess the distribution of ARG hosts, the abundance of ARG hosts was further determined as follows. First, the coverage for each contig was calculated by mapping clean reads from each sample against the assembled contigs using BBMap (https://sourceforge.net/projects/bbmap/) with the parameters “minid = 0.95” and “ambig = random.” Second, the abundance for each contig was determined as the ratio of coverage for each contig to the coverage for all contigs. Finally, the abundance of ARG hosts was obtained according to the taxonomic annotation of ARG-carrying contigs in each sample.

#### Horizontal gene transfer (HGT) analysis

To explore the potential HGT of ARGs among microbes, mobile genetic elements (MGEs) were characterized by annotating all ORFs on ARG-carrying contigs (ACCs) against the NCBI non-redundant (NR) protein database. Annotations were categorized as MGEs based on string matches to each of the following keywords: transposase, transposon, conjugative, integrase, integron, recombinase, conjugal, mobilization, recombination, plasmid, and relaxase [37, 38]. Subsequently, the MGE coverage was normalized by the data size of each sample (copies/Gb) to compare the MGE profile among different samples.

#### Metagenomic binning

Genome binning of each metagenome was performed using MetaWRAP [39]. The completeness and contamination of the MAGs were evaluated using CheckM [40], and high-quality MAGs (completeness > 50%, contamination < 10%) were kept for downstream analysis. The taxonomy of the recovered metagenome-assembled genomes (MAGs) was determined using a set of 120 universal single-copy proteins based on the Genome Taxonomy Database (GTDB) using GTDB-Tk [41]. The amino acid identity (AAI) between MAGs was determined using CompareM with default options (https://github.com/dparks1134/CompareM), and the redundant MAGs were dereplicated with the 99.5% average nucleotide identity (ANI) [42]. The coverage of each MAG was calculated by BBMAP (https://sourceforge.net/projects/bbmap/) with the default parameters.

### Statistical analysis

Principal coordinates analysis (PCoA) was performed to visualize the dissimilarity of ARGs and hosts in all samples based on Bray-Curtis similarity matrices. Analysis of similarity (ANOSIM) was conducted to test the significance of the differences among a priori sampling groups based on environmental parameters. PCoA and ANOSIM statistics were carried out using the *vegan* package in R. Linear discriminant analysis effect size (LEfSe) [43] was used in conjunction with the Kruskal-Wallis and Wilcoxon tests to discover high-dimensional biomarkers and explain the taxa differences for different landform types [44]. One-way analysis of variance (one-way ANOVA) was executed to test the significance of group differences using *vegan*. Sloan et al.’s neutral community model [45] was used to assess the potential importance of neutral processes on the ARG/host/HPB communities. Procrustes analysis was also conducted to assess the correlation between ARGs and their hosts in the Yangtze River by using PCoA results as input. The measure of fit *M*^2^ (the sum of squared distances between matched sample pairs) and *P-*value were computed from 10,000 labeled permutations using *vegan*.

Distance-decay patterns of the similarity of ARG/host/HPB communities were obtained by considering geographical distances from sample site locations to the river mouth. Mantel tests were used to examine the Spearman’s rank correlation between geographical distance and ARG/host/HPB community similarity using Bray-Curtis distance matrices with 999 permutations in R. The geographical distance of each sampling site was calculated using the ArcGIS V10.3 software. The rate of distance-decay of ARG/host/HPB communities was calculated as the slope of the ordinary least-squares regression line fitted to the relationship between geographic distance and community similarity.

Of the driving factors under consideration [21, 22], physicochemical variables included pH, flow discharge (Q), water temperature (Tw), total nitrogen (TN), dissolved oxygen (DO, for water), nitrate-nitrogen (NO_3_^−^-N), ammonium-nitrogen (NH_4_^+^-N), total phosphorus (TP), and total organic carbon (TOC). Spatial variables were determined using principal coordinates of neighbor matrices (PCNM) analysis based on geographic coordinates [46]. Anthropogenic variables, comprising population, GDP, aquatic production, meat production, chemical fertilizer consumption, municipal domestic sewage, the number of patients diagnosed and treated, and the number of residential patients, were determined according to the main administrative region covered by each basin, referred to a previous study [10]. Variables with variance inflation factors (VIF) > 5 were removed using *vegan*. A forward selection step was performed to select variables. A partial least squares-path modeling (PLS-PM) analysis was then conducted to determine the direct and indirect effects of selected variables on ARG/host/HPB communities using the *plspm* package in R [47].

## Results

### Composition profiles of ARGs in the Yangtze River

**Fig. 1:**
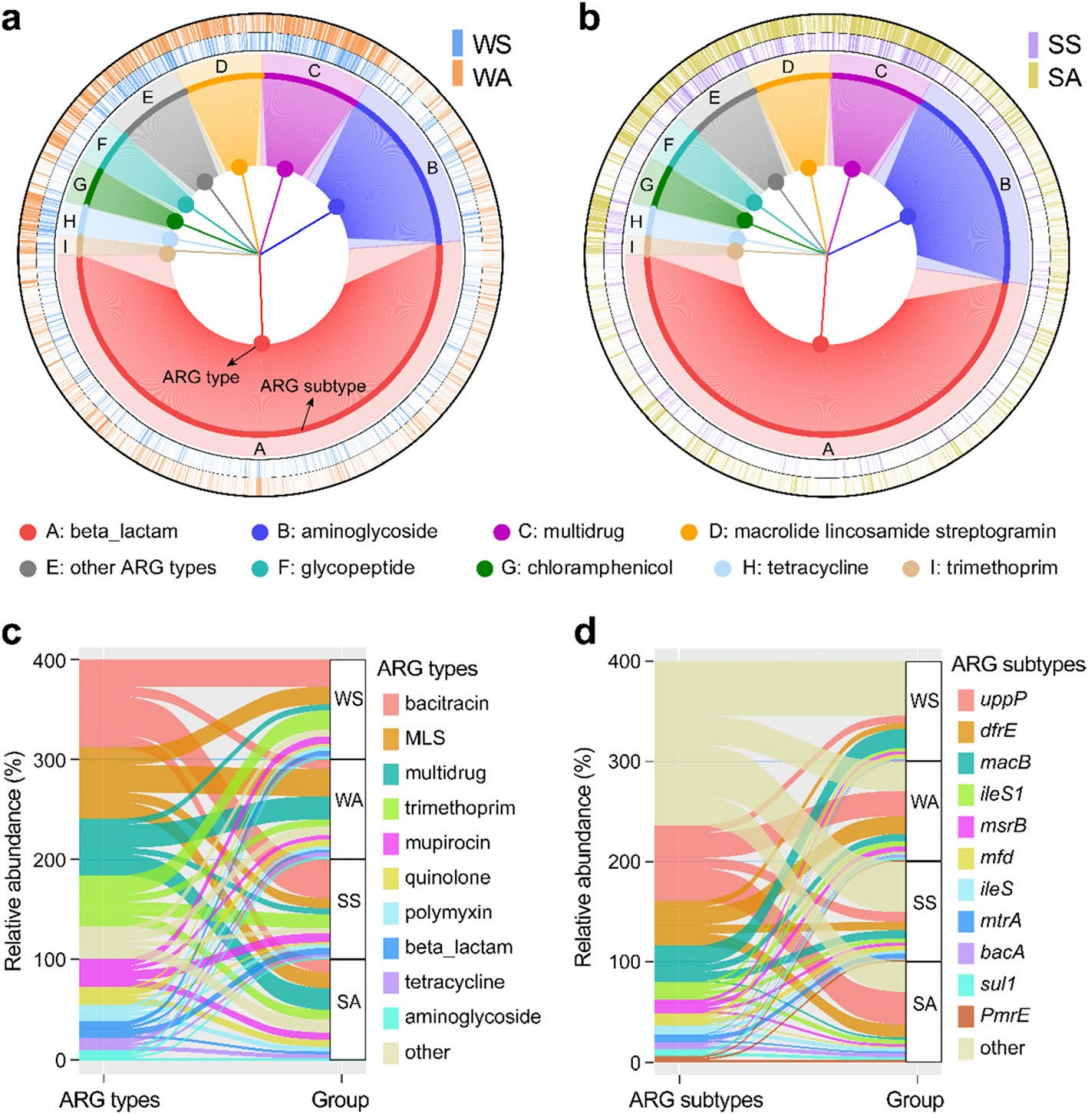
**a** Composition profile of ARG subtypes (inner nodes) and their affiliated ARG types (outer nodes) in water. The transparency of the histogram in the inner and outer rings respectively reflects the occurrence frequency of each ARG subtype in water-spring (WS) and water-autumn (WA) samples. **b** Composition profile of ARG subtypes and their affiliated ARG types in sediment. SS and SA refer to sediment-spring and sediment-autumn samples. **c** Relative abundance of dominant ARG types in each of the four sampling groups. **d** Relative abundance of ARG subtypes in each of the four sampling groups

A broad-spectrum profile of ARGs in the Yangtze River comprised 31 main types and 2195 subtypes, of which the most diverse ARG type was identified as beta-lactam resistance genes containing 1121 subtypes, followed by aminoglycoside, multidrug, macrolide-lincosamide-streptogramin (MLS), glycopeptide, chloramphenicol, tetracycline, and trimethoprim resistance genes (Fig. 1a-b). Moreover, the dominant ARG types included bacitracin resistance genes (relative abundance 22.1%), MLS (17.7%), multidrug (14.2%), and trimethoprim resistance genes (12.7%) (Fig. 1c). Among the ARG subtypes observed in water (89.8% of the total 2195 subtypes) and sediment (85.7%) (Fig. 1d, Fig. S2a-b), the most frequently encountered ARGs were *uppP* (resistant to bacitracin), *drfE* (trimethoprim), and *macB* (MLS) resistance genes (occupancy ≥ 95%) with average abundances of 18.9%, 11.2%, and 9.8%.

The composition of ARG subtypes in different media (water and sediment) and seasons (spring and autumn) was visualized using principal coordinates analysis (PCoA), and four distinct groups were clustered for ARGs (Fig. S2c-d), as confirmed by the ANOSIM test (Fig. S2e-f, ANOSIM *R* > 0.7, *P* = 0.001). Furthermore, sedimentary ARGs exhibited higher richness and diversity than planktonic ARGs, and the ARG compositions were more abundant in autumn (1.79 copies/16S rRNA copies in water and 2.02 in sediment) than in spring (0.310 in water and 0.168 in sediment) (Fig. S3a-b). In terms of seasonal sensitivity of ARGs (Fig. S3c-d), 12 dominant ARG types (e.g., multidrug, MLS, and quinolone resistance genes) prevailed in autumn, whereas 5 ARG types (including resistance to bacitracin, trimethoprim, beta-lactam, sulfonamide, and mupirocin) exhibited higher abundance in spring.

### Taxonomic profiles of ARG hosts

**Fig. 2:**
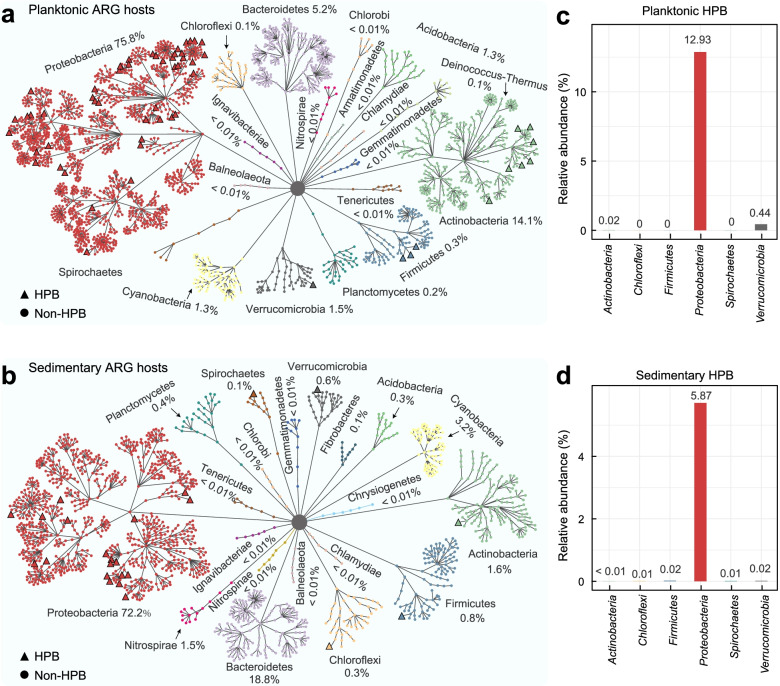
Taxonomic profiles of ARG hosts in water (**a**) and sediment (**b**) of the Yangtze River. Circles and triangles respectively denote human pathogen bacteria (HPB) and other non-HPB ARG hosts. Bar plots indicate the composition of HPB at the phylum level in the water (**c**) and sediment (**d**)

Detectable ARGs in the Yangtze River were taxonomically annotated to a total of 1853 antibiotic-resistant species belonging to 22 phyla. ARG hosts displayed significantly higher biodiversity in water (1408 species) than in sediment (796 species) (Figs. S4-S5, ANOVA *P* < 0.001). Most planktonic ARG hosts were affiliated with Proteobacteria (75.8%), Actinobacteria (14.1%), and Bacteroidetes (5.2%) (Fig. 2a), while sedimentary hosts were mostly associated with Proteobacteria (72.2%) and Bacteroidetes (18.8%) (Fig. 2b). At the genus level, *Limnohabitans*, *Acidimicrobium*, and *Candidatus Methylopumilus* contributed about half of the relative abundance in the planktonic hosts (Fig. 2c), whereas *Methylotenera* and *Flavobacterium* dominated the sedimentary hosts (Fig. 2d, relative abundance 43.90%). In addition, weak seasonal distinctions were observed in both planktonic (Fig. S4c, ANOSIM *R* = 0.244, *P* = 0.001) and sedimentary (Fig. S4d, ANOSIM *R* = 0.086, *P* = 0.001) ARG hosts. In planktonic ARG hosts, the seasonal difference was exhibited by 18 genera, namely *Rhizobacter*, *Andreprevotia*, *Lysobacter*, *Woeseia*, *Vibrio*, *Delftia*, *Nitrosomonas*, *Thiobacillus*, *Knoellia*, *Azospirillum*, *Sphingobium*, *Rheinheimera*, *Porphyrobacter*, *Sphingopyxis*, and *Curvibacter* abundant in spring, and *Synechococcus*, *Cyanobium*, and *Microcystis* in autumn (Fig. S5c). In sedimentary ARG hosts, five genera (including *Elizabethkingia*, *Flavobacterium*, *Rhodoferax*, *Streptomyces*, and unclassified *Comamonadaceae*) exhibited higher abundance in autumn (Fig. S5d).

**Fig. 3:**
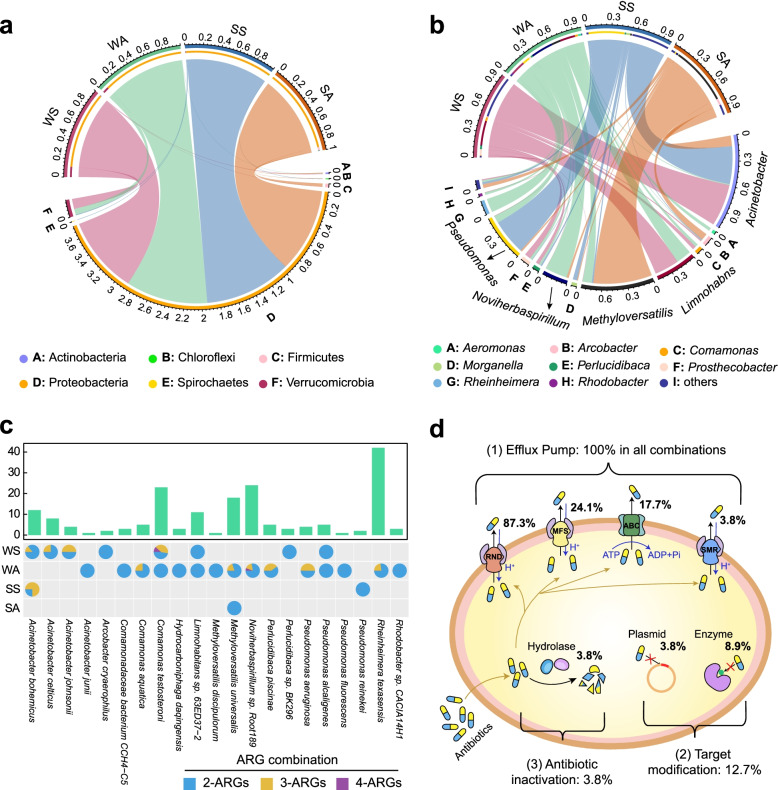
Composition of HPB at the phylum (**a**) and genus (**b**) levels for the four sampling groups in the Yangtze River. **c** HPB carrying ARG combinations of different subtypes, with the labels 2-ARGs, 3-ARGs, and 4-ARGs respectively denoting ARG combinations involving 2, 3, and 4 ARG subtypes. **d** Antibiotic resistance mechanisms for multiple ARG combinations carried by HPB

Specifically, 65 HPB carrying ARGs were identified in water (56 species) and sediment (24 species) of the Yangtze River (Additional file 2: Table S4). Planktonic HPB were dominated by *Limnohabitans* sp. 63ED37-2 (38.9%), *Acinetobacter bohemicus* (14.8%), *Rheinheimera texasensis* (13.0%), *Noviherbaspirillum* sp. Root189 (7.0%), *Methyloversatilis universalis* (5.5%), and *Pseudomonas aeruginosa* (3.6%), while *Methylotenera* sp. G11 (22.3%), *Flavobacterium fluvii* (10.4%), and *Methyloversatilis* sp. RAC08 (3.3%) were the most abundant sedimentary HPB. Although HPB occupied a small percentage of the entire planktonic (13.4%) and sedimentary (5.9%) hosts, they carried 64.4% and 46.1% of the total number of planktonic and sedimentary ARG subtypes, respectively. At the phylum level (Fig. 3a), HPB were mainly affiliated with Proteobacteria in both planktonic HPB (relative abundance 96.5%) and sedimentary HPB (abundance 98.9%). At the genus level (Fig. 3b), *Acinetobacter*, *Limnohabitans*, and *Pseudomonas* were the most common HPB, with a higher average abundance in spring (80% in water, 88% in sediment) than in autumn (28% in water, 14% in sediment). In addition, other dominant HPB genera such as *Comamonas* (4%) and *Prosthecobacter* (7%) prevalent in water_spring, *Methyloversatilis* (11%) and *Rheinheimera* (16%) abundant in water_autumn, *Morganella* (5%) in sediment_spring, and *Methyloversatilis* (70%) prevalent in sediment_autumn.

### Potential horizontal gene transfer of ARGs and ARG hosts

To further evaluate the potential mobility of ARGs, the co-occurrence of specific MGEs and ARGs was investigated. Although many kinds of MGEs were detected to co-localize with ARGs, there were few ACCs (1568) carrying MGEs in the Yangtze River, responsible for 1.23% of all the detected ACCs (127,097). Specifically, the ARGs co-occurring with MGEs, accounting for 0~7.55% of the total ARGs in the river, exhibited a higher abundance in water (average 15.71 coverage/Gb in spring and 17.37 in autumn) than in sediment (2.61 in spring and 1.35 in autumn), demonstrating greater horizontal gene transfer (HGT) potential of ARGs in water (Fig. S6a). Moreover, the most frequently detected MGEs included recombinases, transposases, integrases, and conjugal transfer proteins in both water and sediment (Fig. S6b), and these mobility elements tended to co-exist with multiple ARGs conferring resistance to multidrug, quinolone, and macrolide-lincosamide-streptogramin, thereby promoting the development of multi-antibiotic resistance and the emergence of pathogenic microbes in the river ecosystem. In addition, MGEs were significantly associated with HPB (Additional file 2: Table S5, Spearman’s = 0.48~0.64, *P* < 0.05), followed by ARG hosts (Spearman’s = 0.25~0.65, *P* < 0.05) and ARGs (*P* > 0.05), implying relatively higher environmental and health risks caused by the higher migration and dispersal rates of ARG hosts and HPB along the Yangtze River.

### Relationships between ARGs and bacterial communities

Using the Procrustes analysis based on Bray-Curtis distance, we found significant correlations between ARG profiles and microbial compositions in both water (*M*^2^ = 0.620, *P* < 0.01) and sediment (*M*^2^ = 0.680, *P* < 0.01) (Fig. S7). Unlike previous studies based on co-occurrence relationships [11, 19, 48], we have established direct links between ARGs and their hosts at the phylum and genus levels through metagenomic contigs (Figs. S8-S9). In the planktonic networks (Fig. S9a-b), the direct connections consisted of 159 ARG subtypes and 294 genera in spring and 192 ARGs and 574 genera in autumn. In the sedimentary networks (Fig. S9c-d), such connections occurred between 126 ARG subtypes and 239 genera in spring and 142 ARGs and 381 genera in autumn. These findings indicate that the planktonic hosts tended to carry a greater number of ARGs than the sedimentary hosts, especially in autumn. Complex connections in the networks also occurred between multiple ARGs and HPB in water (Fig. S10). In the planktonic networks, the biggest host nodes including *Methyloversatilis universalis*, *Noviherbaspirillum* sp. Root189, and *Rheinheimera texasensis* connected with more than 40 kinds of ARGs in autumn, while A*cinetobacter bohemicus* and *Acinetobacter celticus* were identified as the biggest nodes (associated with > 31 ARGs) in spring. Among the planktonic HPB, *Comamonas testosteroni* and *Limnohabitans* sp. 63ED37-2 connected with more than 20 kinds of ARGs in both spring and autumn.

We further examined the possibility of multiple ARGs carried by a single HPB. Among the detected 65 HPB, 21 HPB were identified as supercarriers, accounting for 94.4% and 94.5% of the abundance of planktonic and sedimentary HPB, respectively. These 21 supercarriers were found to harbor 79 combinations with multiple ARG subtypes, mainly comprising two kinds of ARGs (56 combinations) (Fig. 3c). Remarkably, planktonic HPB were associated with 97.5% of the total ARG combinations found in the Yangtze, mostly carried by *Rheinheimera texasensis*, *Noviherbaspirillum* sp. Root189, *Comamonas testosteroni*, *Methyloversatilis universalis*, and *Limnohabitans* sp. 63ED37-2 (Fig. 3c). Sedimentary HPB including *Acinetobacter bohemicus*, *Pseudomonas reinekei*, and *Methyloversatilis universalis* served as multiple carriers harboring 6 ARG combinations. Hence, planktonic HPB appeared to contribute far more than sedimentary HPB as supercarriers of ARGs in the Yangtze River. These HPB containing ARG combinations were shaped by three ARG resistance mechanisms (Fig. 3d), namely efflux pump, antibiotic inactivation, and target modification. Antibiotic efflux pumps may be divided into five families [49], namely the major facilitator (MFS) superfamily, the adenosine triphosphate (ATP)-binding cassette (ABC) superfamily, the small multidrug resistance (SMR) family, the resistance-nodulation-cell division (RND) superfamily, and the multidrug and toxic compound extrusion (MATE) family. Surprisingly, each ARG combination encoded at least one antibiotic efflux pump, with 87.3% of the total combinations involved in the resistance-nodulation-division (RND) antibiotic efflux system. In addition, 3.8% and 12.7% of ARG combinations contributed to antibiotic inactivation and target modification mechanisms, respectively.

**Fig. 4:**
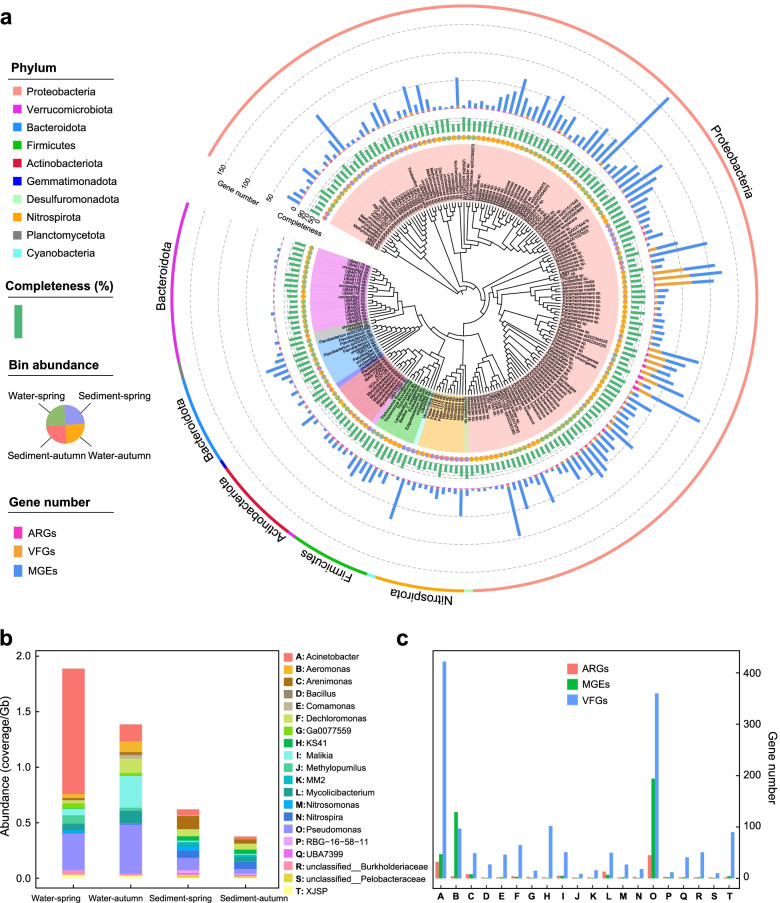
**a** Phylogenetic tree of the retrieved ARG-carrying MAGs in the Yangtze River. **b** Abundance of the multi-antibiotic resistant MAGs (carrying more than two ARGs) at the genus level in the four sampling groups. **c** Gene number of ARGs, MGEs, and VFGs detected in the multi-antibiotic resistant genera

Furthermore, a metagenomic binning analysis was also conducted. A total of 199 metagenome-assembled genomes (MAGs) carrying ARGs were identified in the Yangtze (Additional file 2: Table S6), and these MAGs were assigned to ten phyla among which Proteobacteria (136 MAGs), Verrucomicrobiota (18 MAGs), Bacteroidota (10 MAGs), Actinobacteriota (10 MAGs), and Nitrospirota (10 MAGs) were the dominant phyla (Fig. 4a). The abundance of ARG-carrying MAGs in water-spring (6.69 coverage/Gb) and water-autumn (5.76 coverage/Gb) was significantly higher than that in sediment-spring (1.79 coverage/Gb) and sediment-autumn (1.24 coverage/Gb). The 199 MAGs mainly carried 13 ARG types, among which multidrug, polymyxin, trimethoprim, sulfonamide, and macrolide-lincosamide-streptogramin were most frequently detected. Moreover, 121 ARG-carrying MAGs could be pathogenic ARG hosts due to the disclosure of diverse VFGs (161 types), and these MAGs belonged to *Acinetobacter* (0.46 coverage/Gb), *Malikia* (0.38), *Pseudomonas* (0.23), *Nitrospira* (0.13), *Methylopumilus* (0.12), and *Limnohabitans* (0.10). Strikingly, a wide range of MGEs was frequently detected in these pathogenic resistant bacteria, implying a high HGT potential of ARGs through pathogenic bacteria that further facilitate the dissemination of ARGs and pose genuine threats to human health.

Notably, some bacteria exhibited both possible multi-antibiotic resistance and pathogenicity characteristics. A total of 61 MAGs harbored at least two ARGs and mainly conferred resistance to multidrug, polymyxin, trimethoprim, sulfonamide, and beta-lactam. These MAGs mainly belonged to *Acinetobacter* (average abundance of 0.34 coverage/Gb), *Pseudomonas* (0.23 coverage/Gb), *Planktophila* (0.16 coverage/Gb), and *Malikia* (0.15 coverage/Gb) (Fig. 4b). Interestingly, 77% of the MAGs, mostly annotated as *Acinetobacter*, *Pseudomonas*, and *Malikia*, exhibited a pronounced tendency to carry at least one VFG, suggesting that these environmental bacteria with multiple resistance might have considerable pathogenicity in the river (Fig. 4c). Additionally, these pathogenic multi-resistant bacteria tended to display a higher abundance in water (WS 1.89 coverage/Gb, WA 1.39 coverage/Gb) than in sediment (SS 0.62 coverage/Gb, SA 0.38 coverage/Gb).

### Biogeographic patterns of ARGs and hosts

**Fig. 5:**
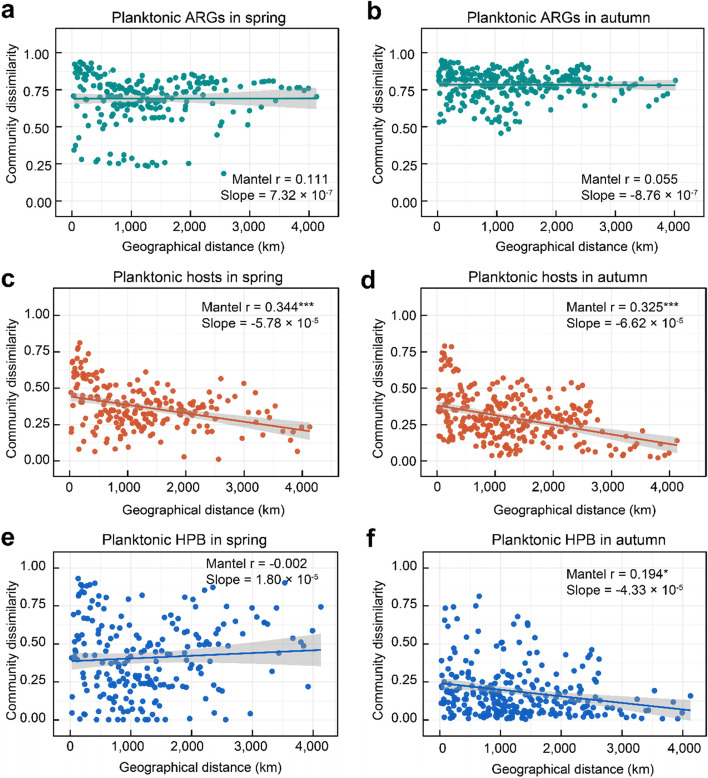
Distance-decay relationships of Bray-Curtis similarity of planktonic ARGs in spring (**a**) and autumn (**b**), hosts in spring (**c**) and autumn (**d**), and HPB with geographical distance in spring (**e**) and autumn (**f**). Mantel-Spearman correlations (*r*) and probabilities (significance codes: ***≤ 0.001, **≤ 0.01, *≤ 0.05) are provided. Solid lines indicate ordinary least squares linear regression across all samples. Slopes of regression lines are also provided

No significant distance-decay was found for the similarity of ARG composition in water and sediment along the Yangtze (Fig. 5a-b and Fig. S11a-b), despite the significant decrease in community similarity of ARG hosts with geographical distance (Fig. 5c-d and Fig. S11c-d, < 0.05). Moreover, the similarity of ARG hosts declined faster in water (with decay slopes of − 5.78 × 10^−5^ in spring and − 6.62 × 10^−5^ in autumn) than in sediment (with decay slopes of − 5.05 × 10^−5^ in spring and − 1.12 × 10^−5^ in autumn), suggesting higher spatial turnover rates of planktonic hosts. Interestingly, both planktonic and sedimentary HPB demonstrated similar mild distance-decay relationships as ARG compositions along the Yangtze (Fig. 5e-f and Fig. S11e-f).

The abundance and distribution of ARGs and their hosts along the mainstream of the Yangtze River were presented, which were further compared with those in the main tributaries such as DTH (Dongtinghu), HBH (Huangbohe), HJ (Hanjiang), JLJ (Jialingjiang), MJ (Minjiang), PYH (Poyanghu), and WJ (Wujiang). The ARGs shared by the mainstream and seven tributaries accounted for 70% and 96% of the total abundance respectively in water and sediment, suggesting similar ARG compositions in most river reaches (Fig. S12, ANOVA *P* > 0.05). Moreover, the seasonal difference in both planktonic and sedimentary ARGs in the domains was characterized by significantly higher richness and abundance in autumn than in spring. Specifically, the genes conferring resistance to multidrug and MLS were higher in autumn, while the bacitracin resistance genes seemed more abundant in spring (Fig. S13).

The spatiotemporal pattern of ARG hosts was also investigated in terms of the mainstream and seven tributaries. No statistical distinction in abundances of ARG hosts was found among mainstream and tributaries (Fig. S14a, ANOVA *P* > 0.05), though significant seasonal differences in ARG hosts existed (ANOVA *P* < 0.05). In particular, planktonic ARG hosts exhibited a significant difference in richness. The mean richness appeared higher in the tributary MJ (81~95) in both spring and autumn, but greater in the mainstream (88) in autumn (Fig. S14b). The planktonic ARG hosts (mainly assigned to Proteobacteria or Actinobacteria) were abundant in tributaries such as WJ, MJ, and HBH in spring (mean abundance 3.10E−3 copies/Gb in WJ, 2.75E−3 in MJ, 1.67E−3 in HBH), while sedimentary ARG hosts (dominated by Proteobacteria) were more abundant in tributaries including HJ (1.50E−3 copies/Gb), DTH (8.31E−4 copies/Gb), and WJ (7.06E−4 copies/Gb) in spring, compared to those in the mainstream (6.21E−4 copies/Gb). In short, ARG hosts exhibited spatiotemporal variations compared with ARGs along the Yangtze River.

Significant differences in the abundance of planktonic HPB were observed in terms of the mainstream and seven tributaries (Fig. S15a, ANOVA *P* < 0.05). In spring, planktonic HPB in the mainstream were dominated by *Limnohabitans* and *Acinetobacter* (mean abundance 1.82E−4 copies/Gb), slightly greater than the tributary HPB, e.g., WJ (1.51E−4 copies/Gb), HBH (1.50E−4 copies/Gb), and MJ (1.27E−4 copies/Gb). In autumn, the highest abundance of HPB occurred at the tributary MJ (4.12E−4 copies/Gb), dominated by *Methyloversatilis* and *Prosthecobacter*, which was considerably higher than those observed in the mainstream (2.14E−4 copies/Gb), dominated by *Limnohabitans*, *Methyloversatilis*, and *Rheinheimera*. In general, planktonic HPB exhibited greater richness in autumn than in spring except for those at tributaries HBH and HJ (Fig. S15b). A similar analysis was made on spatiotemporal distributions of sedimentary HPB (both richness and abundance) in the mainstream and tributaries (Fig. S15). Lastly, planktonic supercarriers as the majority (about 94% of the total abundance) of HPB displayed somehow differences among the mainstream and seven tributaries (Fig. S15c, ANOVA *P* < 0.05) but exhibited significant seasonal differences in tributaries such as WJ and MJ.

Furthermore, landforms may influence the distribution of ARG hosts along the Yangtze River. ANOSIM analysis indicated significant landform difference in planktonic (*R* = 0.376, *P* = 0.002) and sedimentary ARG hosts (*R* = 0.313, *P* = 0.001) in spring. At the genus level, certain ARG hosts (e.g., *Methylotenera* and *Novosphingobium*) were abundant in plain and mountain regions, whereas others (e.g., *Rhodoluna* and *Acidimicrobium*) showed a preference for basin or low hill regions (Fig. S16). However, no distinct difference (ANOSIM *P* > 0.5) occurred in the compositions of planktonic and sedimentary HPB regarding different landforms and none in ARGs except for the river reach with low hill landform (Fig. S17).

### Driving forces for antibiotic resistome

**Fig. 6:**
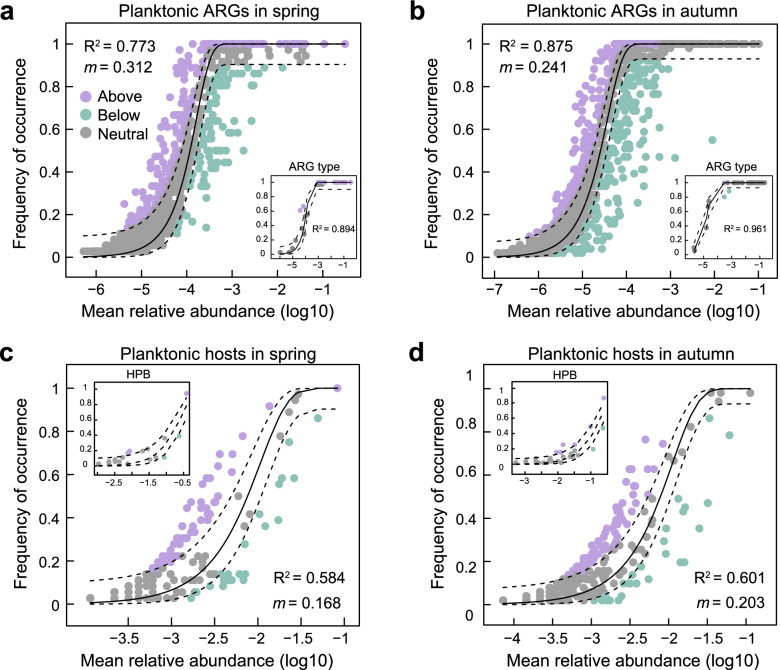
Occurrence frequency of planktonic ARG subtypes in spring (**a**) and autumn (**b**) as well as hosts in spring (**c**) and autumn (**d**) fitted to the mean relative abundance using Sloan et al.’s neutral model [45]. Inserts in (**a**) and (**b**), and (**c**) and (**d**) show neutral model fits to ARG type and HPB. respectively. Purple and green dots indicate ARGs/hosts that occur more (“above”) and less (“below”) frequently than given by the neutral model (gray dots, “neutral”). *R*^2^ indicates the fit to the neutral model, and *m* indicates the immigration rate. Dashed lines represent the 95% confidence intervals about the model prediction

To interpret the ARG profiles in the Yangtze River, we fitted the occurrence frequency of ARGs to a neutral model that incorporated the effects of stochastic dispersal and drift processes. As a result, the neutral process fairly described planktonic ARG subtypes (Fig. 6a-b, spring: *R*^2^ = 0.773; autumn: *R*^2^ = 0.875) and sedimentary ARG subtypes (Fig. S18a-b, spring: *R*^2^ = 0.815; autumn: *R*^2^ = 0.942), as it for ARG types. Non-neutral partitions of ARGs mainly included those with high occurrence frequency, especially genes conferring resistance to aminoglycoside, beta-lactam, MLS, and multidrug (Fig. S19). Compared with ARGs, the ARG hosts showed a slightly poorer fit to the neutral model in water (Fig. 6c, d, spring: *R*^2^ = 0.584; autumn: *R*^2^ = 0.601) and sediment (Fig. S18c-d spring: *R*^2^ = 0.230; autumn: *R*^2^ = 0.109). According to the immigration rate (*m*) estimated from the neutral model, the random dispersal of microbial hosts was likely to be lower than that of ARGs because of multiple ARGs carried by HPB. However, HPB themselves could hardly be described by the neutral model (Fig. 6c-d and Fig. S18c-d) due to the incomprehensive and uncertain number of HPB as putative functional taxa, leading to difficulties in predicting an immigration rate (*m*) for HPB.

**Fig. 7:**
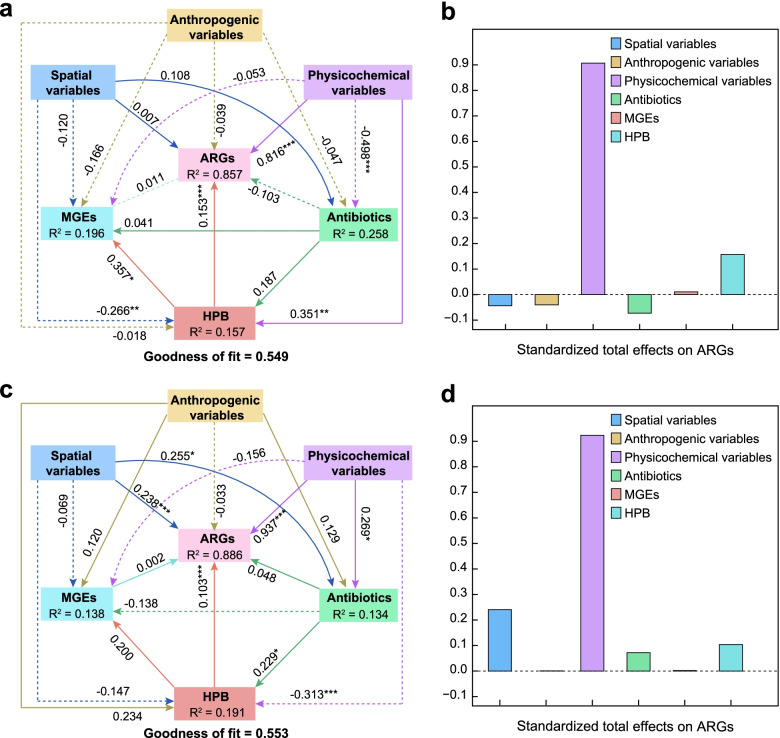
The partial least squares path models showing the effects of spatial variables, anthropogenic variables, physicochemical variables, antibiotics, MGEs, and HPB on ARG compositions in water (**a**) and sediment (**b**) of the Yangtze River. Solid and dashed lines indicate positive and negative effects, respectively. Numbers adjacent to each arrow denote partial correlation coefficients (significance codes: ***≤ 0.001, **≤ 0.01, *≤ 0.05). *R*^2^ values display the proportion of variance explained for each factor. The bar chart showing the standardized total effect of each factor on the ARG composition in water (**c**) and sediment (**d**)

PLS-PM analysis was conducted to assess the direct and indirect effects of spatial variables, anthropogenic variables, physicochemical variables, antibiotics, MGEs, and ARG hosts/HPB on ARG profiles in the Yangtze River. It was found that physicochemical variables represented with water temperature showed the largest positive total standardized effects on the ARG composition in both water and sediment, while spatial variables significantly correlated with sedimentary ARGs (Fig. 7 and Fig. S20). Besides, antibiotics did not show significant direct effects on ARGs in water and sediment but might affect the sedimentary ARG hosts/HPB and thereby indirectly drive the ARG profiles in the river. MGEs seemed to have an insignificant effect on ARGs in both water and sediment, possibly indicating a low HGT frequency of ARG resistome in the Yangtze. However, both ARG hosts and HPB exerted significant effects (path coefficient 0.193~0.818) on MGEs, suggesting that the mobility of the microbes carrying ARGs further promoted the dispersal of ARGs in the water and sediment of the Yangtze River. In addition, anthropogenic variables mainly represented by municipal domestic sewage and the total population had less influence on ARGs, ARG hosts, and HPB in this large natural river. Notably, HPB exhibited positive associations with planktonic and sedimentary ARGs. Compared with the correlations between the richness of microbial hosts and ARGs in water (Fig. S21, Spearman’s *r* = 0.30~0.54, *P* > 0.05), much higher correlations between planktonic HPB and ARGs (Spearman’s *r* = 0.66~0.69, *P* < 0.001) further suggested that planktonic HPB were major contributors to antibiotic resistome in the Yangtze River.

## Discussion

Rivers provide an important route for the propagation of ARGs and pathogens between the environment and human beings. Given the limited information available on the public risk posed by riverine antibiotic resistome, we focused on ARGs and their hosts in water and sediment, paying particular attention to the unique contribution of HPB in determining ARGs along the Yangtze River.

Dominant ARGs captured from the river system (regarded as a “terrestrial gut”) are significantly different from those reported in the human gut, anthropogenic systems, and other systems like lakes or reservoirs. For example, tetracycline resistance genes were ubiquitous and predominant in the human gut worldwide [6]; tetracycline- and aminoglycoside-resistant genes were the most abundant ARG types in sewage treatment plants [50]; multidrug resistance genes, beta-lactamase, and aminoglycoside were the most diverse and dominant ARGs in Xidong Reservoir [51] and estuarine sediments [19]. However, ARGs conferring resistance to bacitracin, MLS, and multidrug resistance genes were found to be predominant in the Yangtze River. Moreover, the microbiota in the human gut typically resist tetracycline through ribosomal protection mechanisms; sewage bacteria primarily resist tetracycline through MFS antibiotic efflux [52]; bacteria in reservoir water [51] and estuarine sediments [19] conferred antibiotic resistances mainly through three resistance mechanisms including antibiotic deactivation, followed by efflux pumps and cellular protection. In comparison, riverine microbial resistance is likely driven by RND efflux pumps. Although ARGs in natural rivers could arise from anthropogenic practices that release antibiotics and/or components of human resistant microbiome, it should be stressed that other sources could include naturally produced antibiotics and resistance genes [53].

ARG hosts may vary in anthropogenic systems and natural rivers. For instance, Firmicutes is the predominant phylum in the human gut [5] and Actinobacteria in polluted farmland [48], whereas the ARG hosts in the Yangtze are similar to the dominant phylum in pristine Antarctic soil [54] given that the majority (> 97%) are taxonomically assigned to Proteobacteria. As ideal pipelines for the transformation and accumulation of ARGs and antibiotic-resistant microbes, river ecosystems could receive resistant microorganisms in water and sediment from basins with different landforms [55]. Besides oligotrophic ultramicrobacteria, such as *Polynucleobacter* and *Limnohabitans* typically observed in freshwater systems [56], other exogenous bacteria also serve as major ARG hosts in the Yangtze. These include *Flavobacterium* as a fish pathogen mainly detected in aquaculture systems [57] and *Methylotenera* and *Acidimicrobium* often discovered in environments affected by intense agricultural activity [58] or metal contamination [59]. Surprisingly, we found that HPB are the predominant multi-antibiotic resistant “supercarriers” along the Yangtze River. Planktonic HPB occupied a small fraction (13.4%) of all ARG hosts but contributed greatly (64.4%) to total ARGs, suggesting the primary importance of HPB in carrying the ARGs in this large river. Metagenomic binning analysis indicated that many unknown environmental bacteria could be potential pathogenic supercarriers of ARGs. For example, HPB, such as *Acinetobacter* spp. and *Pseudomonas* spp., identified to carry at least one VFG, have a remarkable ability to rapidly develop resistance to various antibiotics and persist in the natural environment [60]. Intriguingly, typical HPB in this “terrestrial gut,” including *Limnohabitans* sp. 63ED37-2, *Acinetobacter bohemicus*, *Rheinheimera texasensis*, *Noviherbaspirillum* sp. Root189, and *Methyloversatilis universalis* were significantly different from those represented by *Escherichia coli*, *Klebsiella pneumoniae*, *Staphylococcus aureus*, *Enterococcus fecalis*, *Enterococcus faecium*, and *Pseudomonas putida*, in human/animal guts or sewage systems [33, 61–63].

The multi-resistance of HPB can be further explained with RND efflux pumps [49] which facilitate transporting a variety of antibiotics out of a given cell to confer multidrug resistance and play a predominant role in mediating the cross-resistance of HPB (Fig. 3d). Meanwhile, MFS, ABC, SMR, and MATE efflux systems also contributed to the ARG accumulation of HPB because of their resistance to specific antibiotics such as tetracycline, macrolide, aminocoumarin, and fluoroquinolone. Besides, antibiotic resistance of HPB could be caused by a target modification mechanism, mainly driven by the emergence of *chrB* and *dfrE* genes with resistance to macrolides and trimethoprim through methylation of 23S ribosomal RNA or the production of alternative proteins [64, 65]. Although antibiotic inactivation is known to be prevalent in environments containing many residual antibiotics due to human activities [52], only a few HPB-carrying ARGs such as *APH(3′)-la* may produce specific enzymes that inactivate antibiotics in the Yangtze, indicating a weak contribution of antibiotic inactivation to the multiple resistance of HPB. The co-existence of genes encoding multiple efflux pumps, target modification, and antibiotic inactivation mechanisms promote broad-spectrum resistance in HPB [66]. Dominant HPB such as *Rheinheimera texasensis*, *Methyloversatilis universalis*, and *Commanonas testosteroni* are typical Gram-negative bacteria, which may cause drug-resistant infections which are of significant concern nowadays owing to there being fewer effective antibiotics that target Gram-negative bacteria [67]. Therefore, the widespread distribution of HPB with multiple antibiotic resistance along natural rivers poses a new challenge for the treatment of numerous infections using the existing arsenal of drugs.

The presence of terrestrial HPB is helpful in understanding the spatiotemporal distributions of ARGs and their hosts over the 4300 km continuum of the Yangtze River. Terrestrial HPB indicates the HPB from the terrestrial sources (rather than from the human/animal gut or sewage), mostly from the non-point sources accompanied with water and soil loss in the Yangtze River basin. Seasonal fluctuations in precipitation and temperature would alter runoff and sediment flux and thereby planktonic/sedimentary antibiotic-resistant microorganisms from terrestrial sources in the Yangtze. Higher temperature and increased precipitation in the wet season promote the transmission of ARGs and accelerate the reproduction of ARG hosts [68], leading to an increased abundance of ARGs. Meanwhile, non-point sources, especially from surrounding agriculture and aquaculture activities, could introduce more diverse ARGs and pathogens into rivers with the help of more frequent surface runoff events in the wet season [69, 70]. Spatially, both ARG hosts and bacterial communities exhibit significant distance-decay patterns in the Yangtze [21], whereas the distance-decay is not significant for ARGs in the same river. The unexpected inconsistency between ARGs and their hosts may be interpreted by considering the special role of HPB in the Yangtze. Since HPB appear as the minority of the overall microbial community, their impacts on the spatial dispersal of the entire ARG host community are greatly restricted. On the other hand, the weak distance-decay of HPB as the ARG supercarriers would considerably influence the geographical pattern for ARGs. Such insignificant ARG distance-decay relationships were also observed in other ecosystems, such as those in the natural wetlands across the Qinghai-Tibetan Plateau [71], in agricultural soils across China [72], and in the phyllosphere across eastern and northern Australia [73]. By contrast with the disconnected waterbodies and soils [10, 74], the highly connected river continuum enables ARGs to spread in the streamflow direction [75], thereby weakening the dispersal limitation and the distance-decay of ARGs along the Yangtze.

The prevalence of antibiotic-resistant genes and their microbial hosts has become one of the global challenges in river health maintenance. Riverine ARGs could come from the selection process exerted by accumulated antibiotics, or from ARMs released from wastewater or soil runoff. Generally, the majority of input antibiotics are easily degraded and non-persistent with low concentrations in receiving natural rivers, providing a low likelihood of stimulating the prevalence of ARGs [76]. This speculation is consistent with the weak correlations between antibiotics and ARGs (Fig. 7 and Fig. S20). On the other hand, the convergence of resistant microorganisms from multiple terrestrial sources entering rivers may cause persistent ARG pollution. Unlike conventional feces-related bacteria in severely contaminated urban rivers [77], terrestrial HPB as supercarriers have a direct influence on the distribution and dissemination of ARGs in the Yangtze. Here, non-point source (NPS) systems possibly became the main contamination sources of nutrients, harmful substances, and even pathogenic bacteria to the receiving river, through surface runoff, soil erosion, and farmland drainage [78, 79]. Therefore, priority should be given to the prevention of terrestrial HPB from NPS contamination. Besides the reasonable utilization of low-antibiotic drugs and fertilizers, continuous supervision and land-use optimization would also be helpful in reducing the consumption of antibiotics and the accumulation of terrestrial HPB in river ecosystems [80].

To prevent the dissemination of potential resistant HPB from NPS systems into surrounding rivers, integrated management is required that incorporates HPB regulation within effective countermeasures for water and soil conservation. Engineering measures such as constructions of sand block retaining buildings and artificial wetland plants are proposed to stabilize channel slopes and reduce the entry of terrestrial HPB into rivers. Alternative strategies such as the introduction of cropland buffers or additional vegetation cover would also be useful to intercept surface runoff [70]. Noting the high capability of certain HPB like *Acinetobacter* spp. and *Pseudomonas* spp. to develop antibiotic resistance in the natural environment, mitigation strategies are required to reduce the spread of terrestrial HPB during high river flow and in the wet season. Furthermore, collection facilities (such as sewage ponds) are necessary to centralize the disposal of antibiotics and resistant HPB in runoff. To avoid the transfer of resistant HPB into other environmental compartments and to human individuals, novel treatment technologies (such as ultraviolet irradiation, chlorination, and sonication) should be implemented to sterilize water used for irrigation, farming, and drinking [81]. Given the new challenge of “natural” supercarriers, water authorities must pay special attention to terrestrial HPB when developing future strategies for controlling ARGs in large river basins.

## Conclusions

Based on the derived ARGs (31 main types and 2,195 subtypes) and their hosts (22 phyla and 1853 species), we revealed the significant role of HPB in carrying ARGs in water and sediment over a 4300-km continuum along the Yangtze River, the third longest river in the world. Despite their small share (13.4%) in ARG hosts, HPB harbored multiple ARGs accounting for 64% of the total ARGs in water. Multiple resistance of HPB was promoted by resistance-nodulation-cell division (RND) efflux pumps that predominated in 79 ARG combinations. Unlike conventional supercarriers (e.g., *Enterococcus* spp. and other fecal indicator bacteria) prevalent in the human gut, we found that HPB are characterized by “natural” supercarriers (e.g., *Rheinheimera texasensis* and *Noviherbaspirillum* sp. Root189) from the river catchments. From a metagenomic perspective, HPB acting as a key reservoir of ARGs in the complex “terrestrial gut” helped interpret the inconsistency between spatial dissimilarities in ARGs and their hosts and highlighted the importance of HPB-dependent strategies in ARG-related risk management of large rivers globally.

## Supplementary Information

**Additional file 1: Figure S1**. Map of sampling sites in the Yangtze River covering the actual sinuous channel reach of length 4,300 km (equivalent to 2.05 times the 2,102 km straight line joining the start to end sampling sites). **Figure S2**. Venn diagrams showing unique and shared numbers of ARG types (a) and subtypes (b) among the water-spring (WS), water-autumn (WA), sediment-spring (SS), and sediment-autumn (SA) samples in the Yangtze River. Principal coordinates analysis (PCoA) diagrams showing the compositional dissimilarity of ARG type (c) and subtypes (d) in the Yangtze River. Analysis of similarity statistics (ANOSIM) displaying the dissimilarities of ARG subtypes in water (e) and sediment (f) between different seasons. **Figure S3**. Richness (a) and Shannon diversity (b) of ARG subtypes in each sample group of the Yangtze River. ARG types exhibiting significant seasonal differences in water (c) and sediment (d). **Figure S4**. (a) Principal coordinates analysis (PCoA) showing compositional dissimilarities (Bray-Curtis) of ARG hosts (at the species level) in the four sample groups of the Yangtze River. (b-d) ANOSIM statistics concerning differences in ARG hosts within and between sample groups. **Figure S5**. Richness (a) and Shannon diversity (b) of ARG hosts in each sample group of the Yangtze River. ARG hosts (at the species level) exhibiting significant seasonal differences in water (c) and sediment (d). **Figure S6**. (a) Distribution of MGEs co-existing with ARGs across four sampling groups. (b) Distribution of various types of MGEs (co-occurring with ARGs) across four sampling groups. **Figure S7**. Procrustes analysis depicting correlations between ARG subtypes and ARG hosts in water (a) and sediment (b). Solid and hollow circles represent ARGs and hosts, respectively. **Figure S8**. Relationships between ARG hosts at the phylum level (inner circle) and ARG types (outer circle) in water-spring (a), water-autumn (b), sediment-spring (c), and sediment-autumn samples (d). **Figure S9**. Networks displaying ARG hosts carrying multiple ARGs in water-spring (a), water-autumn (b), sediment-spring (c), and sediment-autumn samples (d) of the Yangtze River. The sizes of nodes correspond to the connection degree. **Figure S10**. Networks displaying HPB carrying multiple ARGs in water-spring (a), water-autumn (b), sediment-spring (c), and sediment-autumn samples (d) of the Yangtze River. The sizes of nodes correspond to the connection degree. **Figure S11**. Distance-decay relationships of Bray-Curtis similarity of sedimentary ARGs in spring (a) and autumn (b), hosts in spring (c) and autumn (d), and HPB with the geographical distance in spring (e) and autumn (f). Mantel-Spearman correlations (r) and probabilities (significance codes: ***≤ 0.001 **≤ 0.01 *≤ 0.05) are provided. Solid lines indicate the ordinary least squares linear regression across all samples. Slopes of regression lines are also provided. **Figure S12**. (a) Abundance (copy of ARG per copy of 16S-rRNA gene) and (b) richness of planktonic and sedimentary ARG subtypes in mainstream and seven tributaries (DTH: Dongtinghu, HBH: Huangbohe, HJ: Hanjiang, JLJ: Jialingjiang, MJ: Minjiang, PYH: Poyanghu, and WJ: Wujiang) along the Yangtze River. WS, WA, SS, and SA refer to water-spring, water-autumn, sediment-spring, and sediment-autumn samples, respectively. **Figure S13**. (a) Abundance (copy of ARG per copy of 16S-rRNA gene) and (b) richness of planktonic and sedimentary ARG types in mainstream and seven tributaries (DTH: Dongtinghu, HBH: Huangbohe, HJ: Hanjiang, JLJ: Jialingjiang, MJ: Minjiang, PYH: Poyanghu, and WJ: Wujiang) along the Yangtze River. WS, WA, SS, and SA refer to water-spring, water-autumn, sediment-spring, and sediment-autumn samples, respectively. **Figure S14**. (a) Abundance (coverage/Gb) and (b) richness of planktonic and sedimentary ARG hosts in mainstream and seven tributaries (DTH: Dongtinghu, HBH: Huangbohe, HJ: Hanjiang, JLJ: Jialingjiang, MJ: Minjiang, PYH: Poyanghu, and WJ: Wujiang) along the Yangtze River. WS, WA, SS, and SA refer to water-spring, water-autumn, sediment-spring, and sediment-autumn samples, respectively. **Figure S15**. (a) Abundance (coverage/Gb) and (b) richness of planktonic and sedimentary HPB in mainstream and seven tributaries (DTH: Dongtinghu, HBH: Huangbohe, HJ: Hanjiang, JLJ: Jialingjiang, MJ: Minjiang, PYH: Poyanghu, and WJ: Wujiang) along the Yangtze River. (c) Abundance (coverage/Gb) of planktonic and sedimentary supercarriers in mainstream and seven tributaries. WS, WA, SS, and SA refer to water-spring, water-autumn, sediment-spring, and sediment-autumn samples, respectively. **Figure S16**. LEfSe cladogram depicting the taxonomic differences of ARG hosts in water (a) and sediment (b) in spring for four landform types. Differentially abundant taxa (biomarkers) are colored according to their most abundant landform habitats. **Figure S17**. ARG types (water-spring group) exhibiting significant differences among four landform types along the Yangtze River. **Figure S18**. Occurrence frequency of sedimentary ARG subtypes in spring (a) and autumn (b) as well as hosts in spring (c) and autumn (d) fitted to mean relative abundance using Sloan *et al*.’s neutral community model. Inserts in (a-b) and (c-d) show the neutral community model fits to ARG type and HPB. Purple and green dots indicate ARGs/hosts that occur more (‘Above’) and less (‘Below’) frequently than given by the neutral model (gray dots, ‘Neutral’). R^2^ indicates the fit to the neutral community model, and *m* indicates the immigration rate. Dashed lines represent 95% confidence intervals about the model prediction. **Figure S19**. Distribution of occurrence frequency (a) and mean relative abundance (b) of ARGs in different sampling groups. ‘Above’ and ‘Below’ indicate ARGs that occur more and less frequently than given by the neutral model (‘Neutral’). **Figure S20**. The partial least squares path models showing the effects of spatial variables, anthropogenic variables, physicochemical variables, antibiotics, MGEs, and ARG hosts on ARG compositions in water (a) and sediment (b) of the Yangtze River. Solid and dashed lines indicate positive and negative effects, respectively. Numbers adjacent to each arrow denote partial correlation coefficients (significance codes: ***≤ 0.001 **≤ 0.01 *≤ 0.05). R^2^ values display the proportion of variance explained for each factor. The bar-chart showing the standardized total effect of each factor on the ARG composition in water (c) and sediment (d). **Figure S21**. Spearman’s correlations (R) between the richness of ARG host and ARGs in water (a) and sediment (b), and between the richness of HPB and the richness of ARGs in water (c) and sediment (d). Ordinary least square linear regressions and 95% confidence intervals are also displayed.

**Additional file 2: Table S1**. Detailed information on the 219 samples. **Table S2**. The averaged concentrations of antibiotics measured from four representative sampling groups of the Yangtze River. **Table S3**. Statistics of raw reads and clean reads in each metagenomic dataset. **Table S4**. The list of Human Pathogen Bacteria (HPB) identified in the Yangtze River. **Table S5**. Spearman’s correlations between ARGs, ARG hosts, and mobile genetic elements (MGEs) in the Yangtze River. WS, WA, SS, and SA refer to water-spring, water-autumn, sediment-spring, and sediment-autumn samples. **Table S6**. Detailed information of retrieved MAGs carrying ARGs in the Yangtze River.

## Data Availability

Complete datasets supporting the findings of this article are available in the NCBI Sequence Read Archive (SRA) database (BioProject number: PRJNA559231).
