# Supplementary material for: Supercarriers of antibiotic resistome in a world’s large river
Source: Microbiome. 2022 Jul 28;10:111. doi: 10.1186/s40168-022-01294-z (PMC9331799; doi:10.1186/s40168-022-01294-z)
Supplement: Supplementary file 2 — Additional file 1: Figure S1. Map of sampling sites in the Yangtze River covering the actual sinuous channel reach of length 4,300 km (equivalent to 2.05 times the 2,102 km straight line joining the start to end sampling sites). Figure S2. Venn diagrams showing unique and shared numbers of ARG types (a) and subtypes (b) among the water-spring (WS), water-autumn (WA), sediment-spring (SS), and sediment-autumn (SA) samples in the Yangtze River. Principal coordinates analysis (PCoA) diagrams showing the compositional dissimilarity of ARG type (c) and subtypes (d) in the Yangtze River. Analysis of similarity statistics (ANOSIM) displaying the dissimilarities of ARG subtypes in water (e) and sediment (f) between different seasons. Figure S3. Richness (a) and Shannon diversity (b) of ARG subtypes in each sample group of the Yangtze River. ARG types exhibiting significant seasonal differences in water (c) and sediment (d). Figure S4. (a) Principal coordinates analysis (PCoA) showing compositional dissimilarities (Bray-Curtis) of ARG hosts (at the species level) in the four sample groups of the Yangtze River. (b-d) ANOSIM statistics concerning differences in ARG hosts within and between sample groups. Figure S5. Richness (a) and Shannon diversity (b) of ARG hosts in each sample group of the Yangtze River. ARG hosts (at the species level) exhibiting significant seasonal differences in water (c) and sediment (d). Figure S6. (a) Distribution of MGEs co-existing with ARGs across four sampling groups. (b) Distribution of various types of MGEs (co-occurring with ARGs) across four sampling groups. Figure S7. Procrustes analysis depicting correlations between ARG subtypes and ARG hosts in water (a) and sediment (b). Solid and hollow circles represent ARGs and hosts, respectively. Figure S8. Relationships between ARG hosts at the phylum level (inner circle) and ARG types (outer circle) in water-spring (a), water-autumn (b), sediment-spring (c), and sediment-autumn samples (d). Figur [file 40168_2022_1294_MOESM1_ESM.docx]

**Supplementary Material for**

**Supercarriers of** **antibiotic resistome in a world’s large river**

Jiawen Wang^1,2^, Rui Pan^1,2^, Peiyan Dong^2^, Shufeng Liu^1^, Qian Chen^1,3^, [Alistair G. L. Borthwick](http://pubs.acs.org/action/doSearch?ContribStored=Borthwick%2C+A+G+L)^4,5^, Liyu Sun^1,6^, Nan Xu^6^, Jinren Ni^1^**^*^**

**Author affiliations**:

^1^College of Environmental Sciences and Engineering, Peking University; Key Laboratory of Water and Sediment Sciences, Ministry of Education, Beijing 100871, P. R. China

^2^State Environmental Protection Key Laboratory of All Material Fluxes in River Ecosystems, Beijing 100871, P. R. China

^3^State Key Laboratory of Plateau Ecology and Agriculture, Qinghai University, Xining 810016, P. R. China

^4^Institute of Infrastructure and Environment, School of Engineering, The University of Edinburgh, The King’s Buildings, Edinburgh EH9 3JL, UK

^5^School of Engineering, Computing and Mathematics, University of Plymouth, Drake Circus Plymouth, PL4 8AA, UK

^6^School of Environment and Energy, Peking University Shenzhen Graduate School, Shenzhen 518055, P. R. China

**^*^Correspondence:** Jinren Ni, College of Environmental Sciences and Engineering, Peking University, Beijing 100871, P. R. China. E-mail: jinrenni@pku.edu.cn


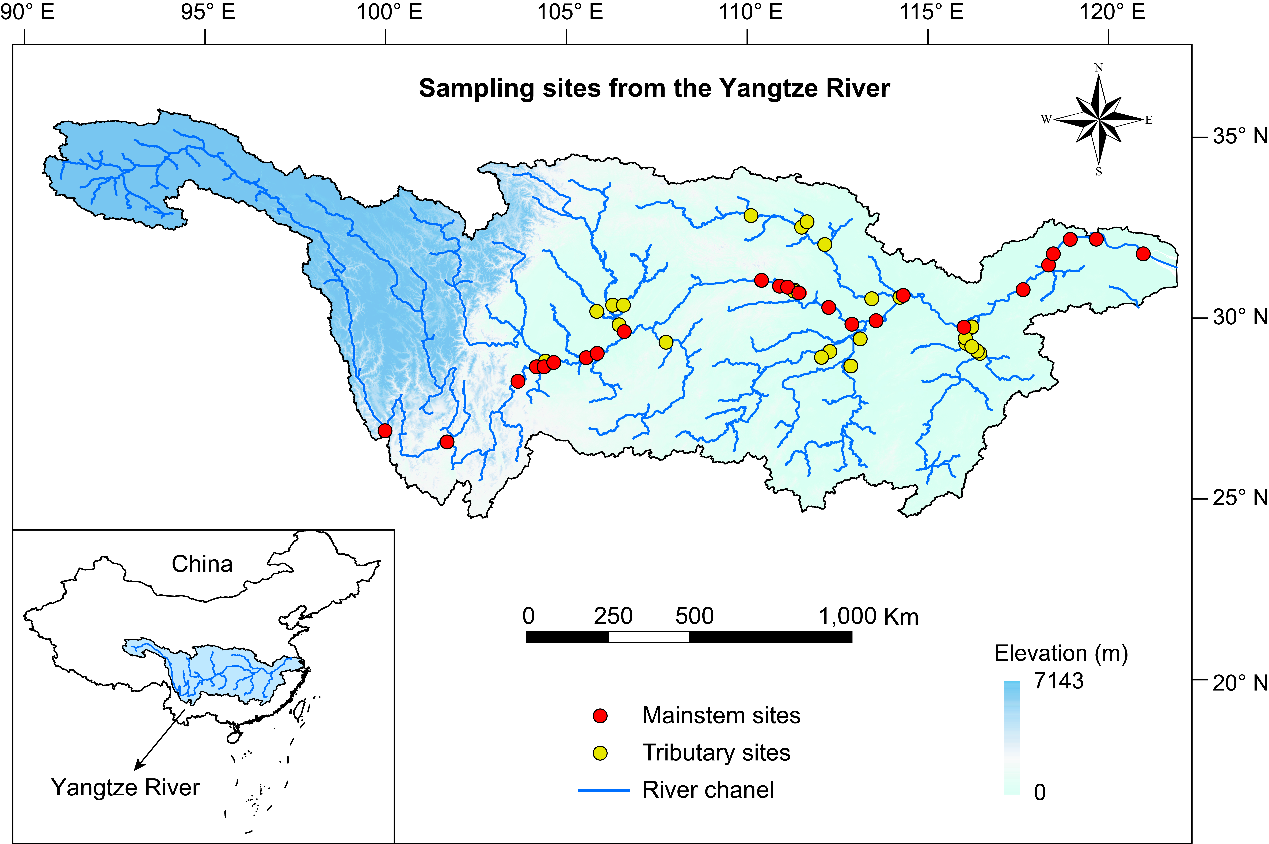


**Figure S1.** Map of sampling sites in the Yangtze River covering the actual sinuous channel reach of length 4,300 km (equivalent to 2.05 times the 2,102 km straight line joining the start to end sampling sites).


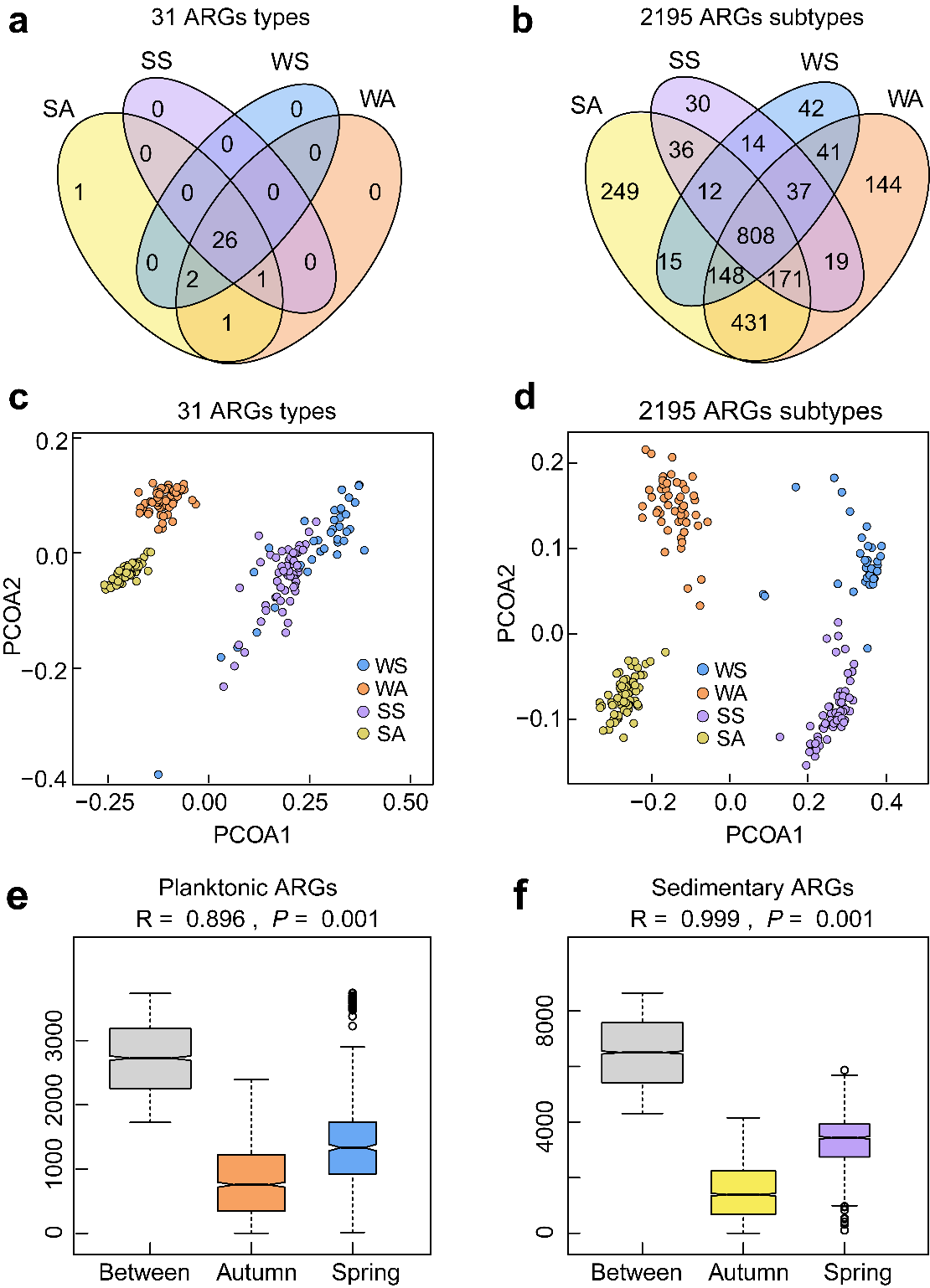


**Figure S2.** Venn diagrams showing unique and shared numbers of ARG types (**a**) and subtypes (**b**) among the water-spring (WS), water-autumn (WA), sediment-spring (SS), and sediment-autumn (SA) samples in the Yangtze River. Principal coordinates analysis (PCoA) diagrams showing the compositional dissimilarity of ARG type (**c**) and subtypes (**d**) in the Yangtze River. Analysis of similarity statistics (ANOSIM) displaying the dissimilarities of ARG subtypes in water (**e**) and sediment (**f**) between different seasons.


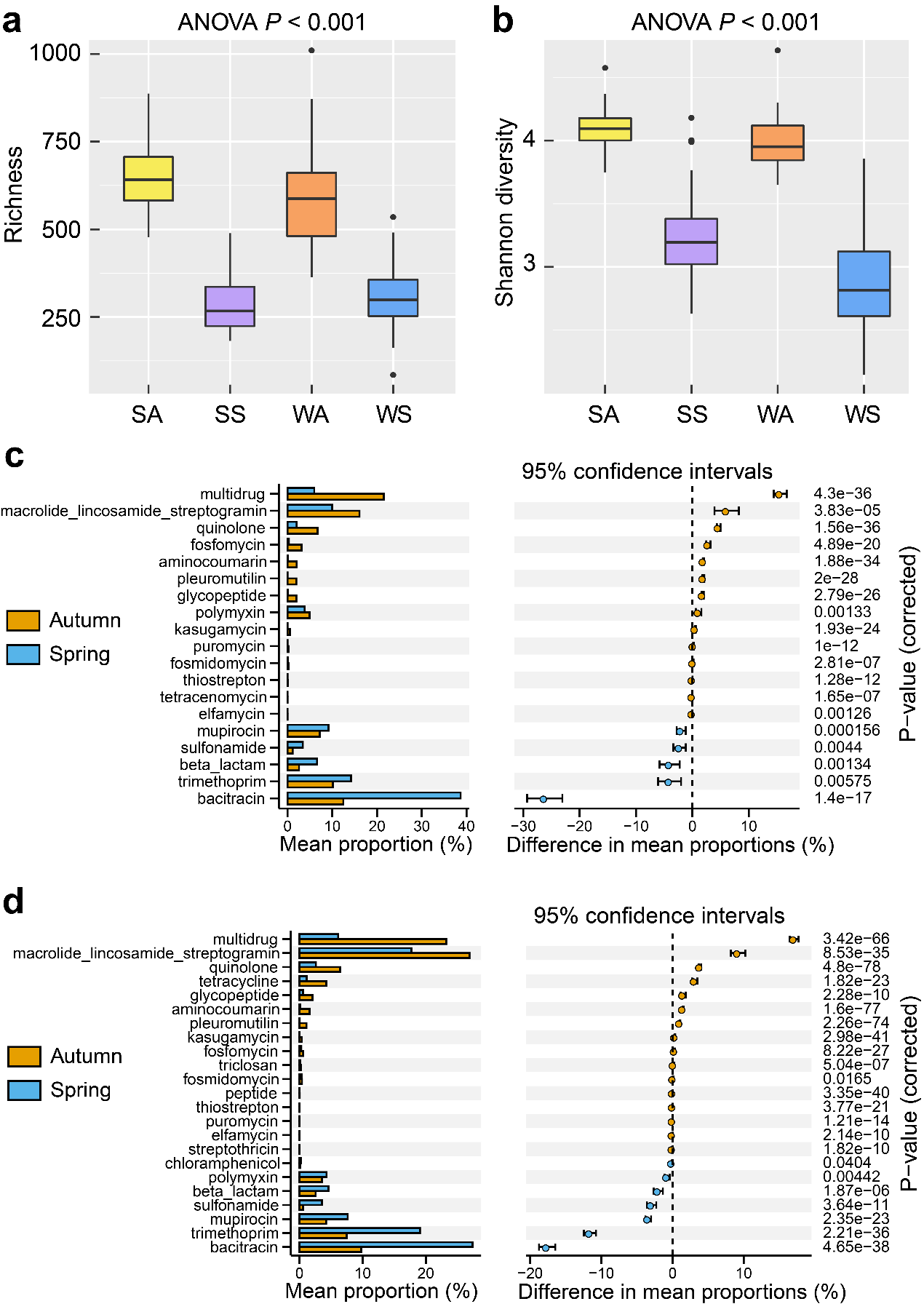


**Figure S3.** Richness (**a**) and Shannon diversity (**b**) of ARG subtypes in each sample group of the Yangtze River. ARG types exhibiting significant seasonal differences in water (**c**) and sediment (**d**).


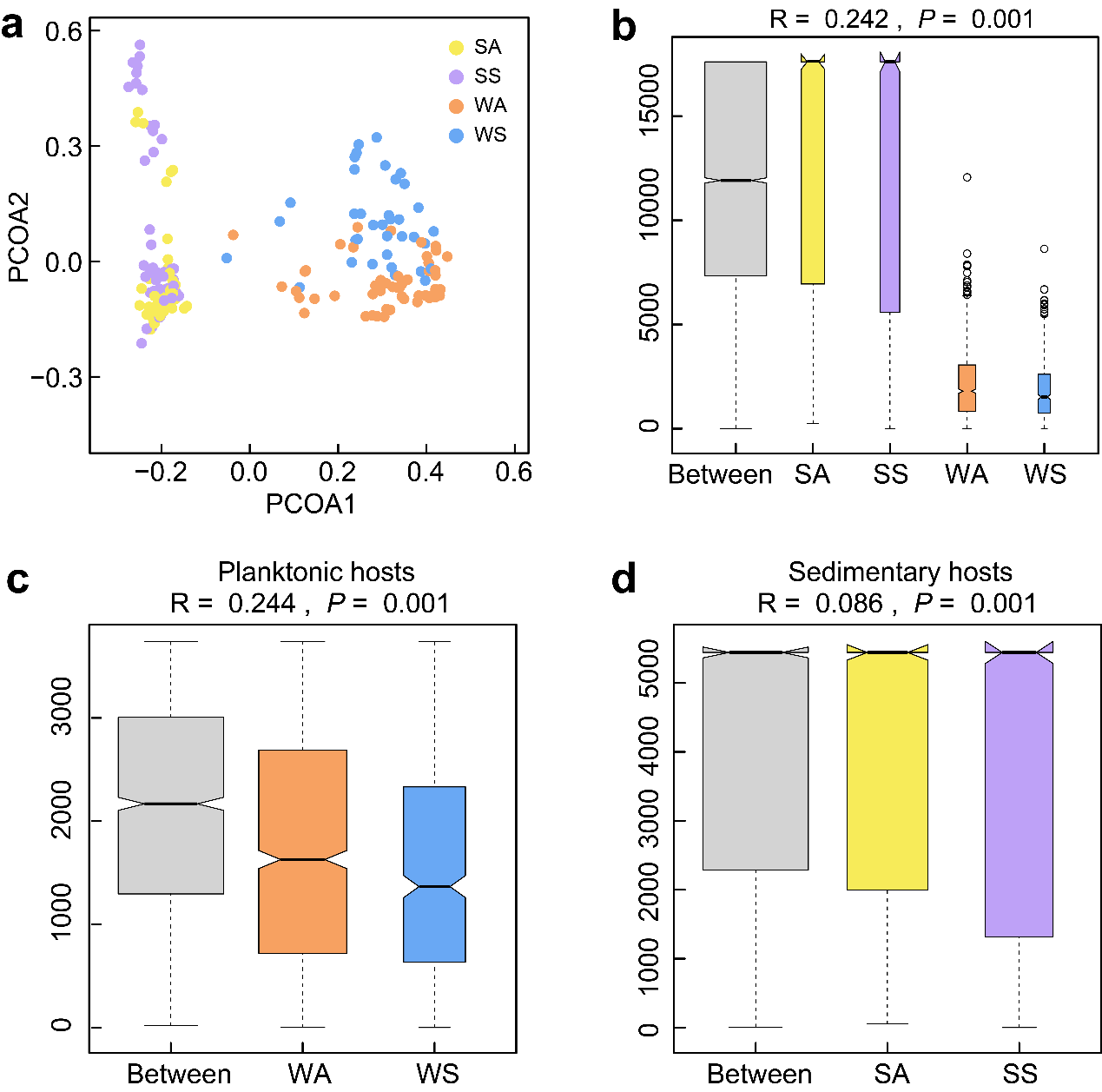


**Figure S4.** (**a**) Principal coordinates analysis (PCoA) showing compositional dissimilarities (Bray-Curtis) of ARG hosts (at the species level) in the four sample groups of the Yangtze River. (**b-d**) ANOSIM statistics concerning differences in ARG hosts within and between sample groups.


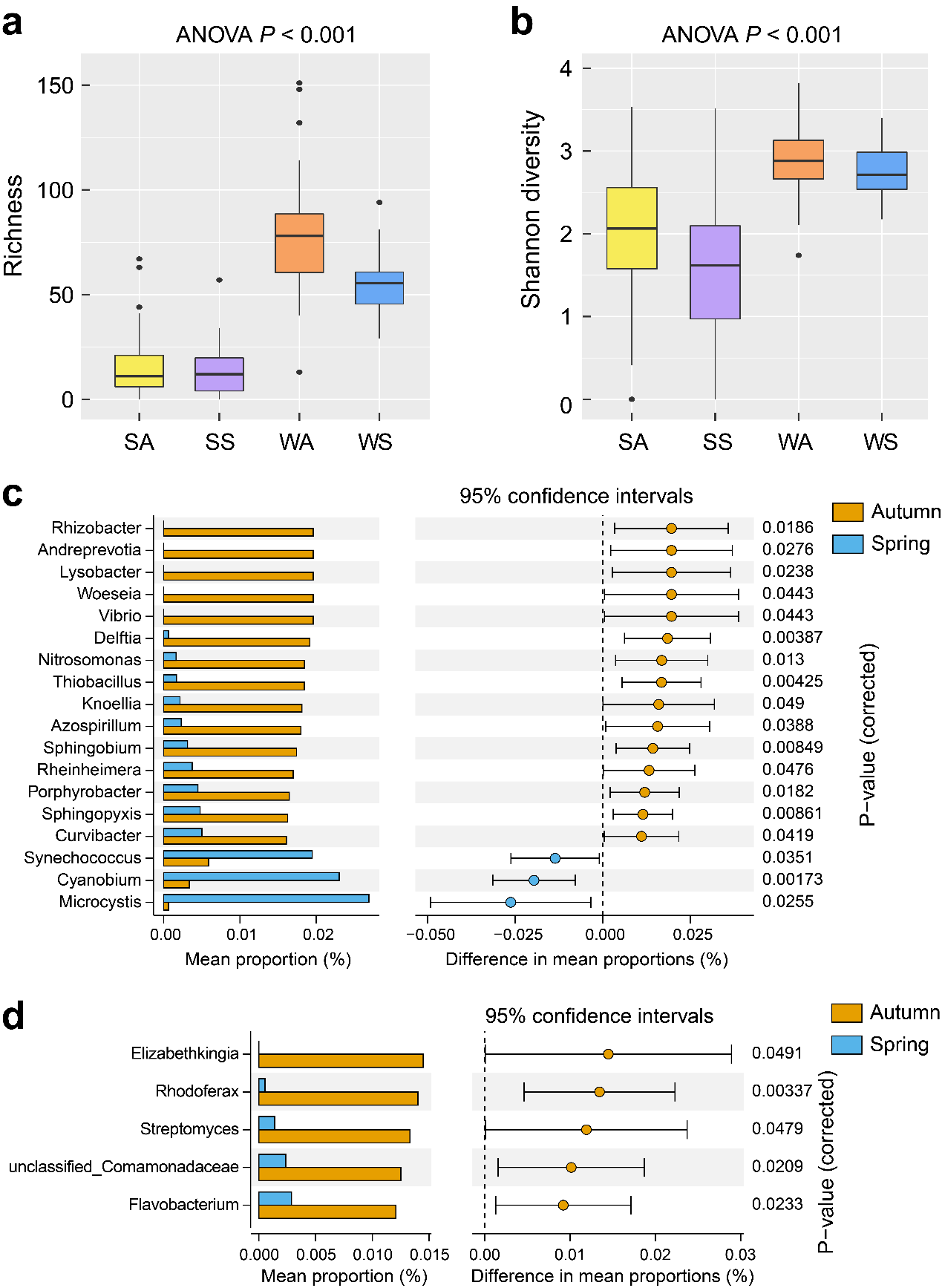


**Figure S5.** Richness (**a**) and Shannon diversity (**b**) of ARG hosts in each sample group of the Yangtze River. ARG hosts (at the species level) exhibiting significant seasonal differences in water (**c**) and sediment (**d**).


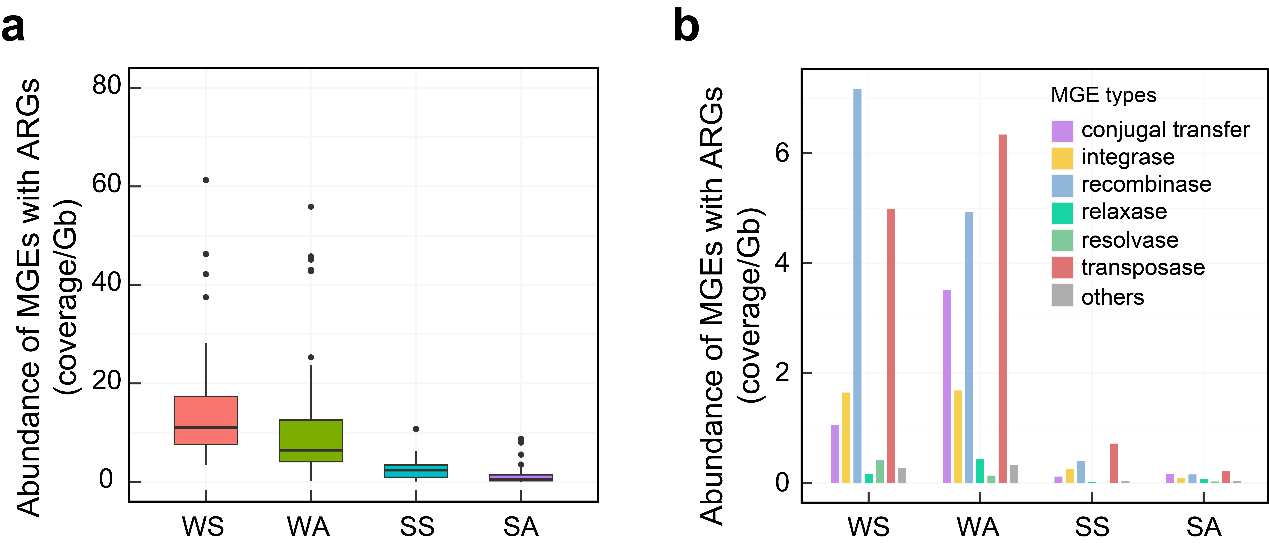


**Figure S6. (a)** Distribution of MGEs co-existing with ARGs across four sampling groups. **(b)** Distribution of various types of MGEs (co-occurring with ARGs) across four sampling groups.


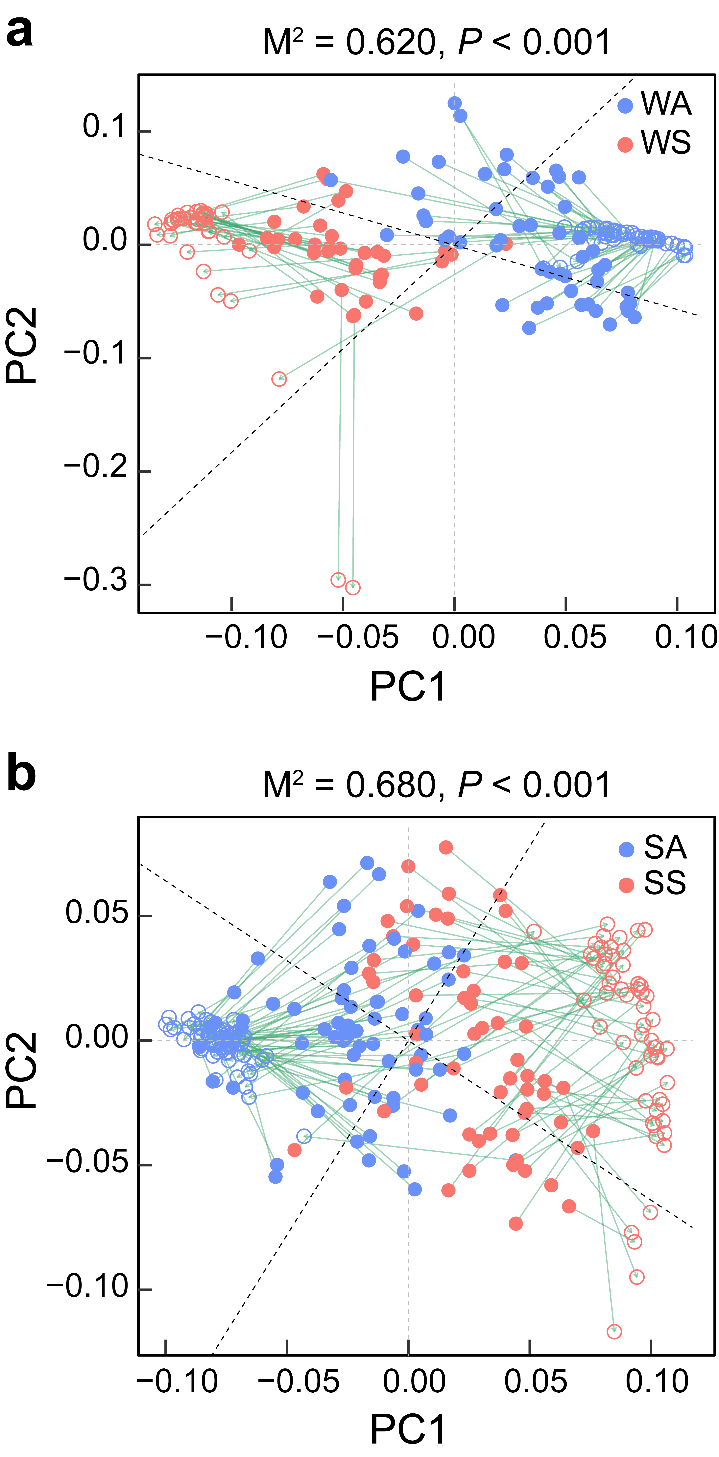


**Figure S7.** Procrustes analysis depicting correlations between ARG subtypes and ARG hosts in water (**a**) and sediment (**b**). Solid and hollow circles represent ARGs and hosts, respectively.


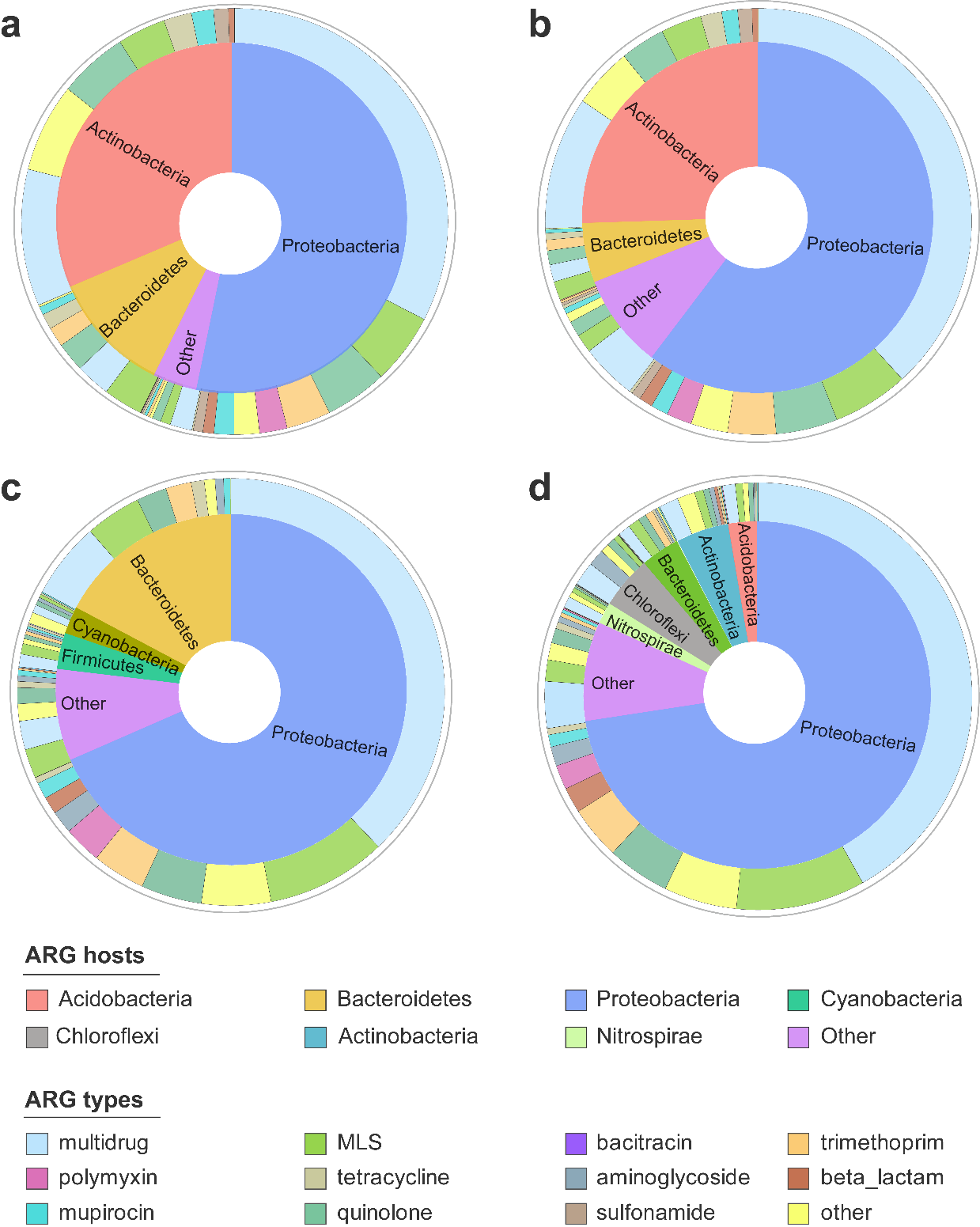


**Figure S8.** Relationships between ARG hosts at the phylum level (inner circle) and ARG types (outer circle) in water-spring (**a**), water-autumn (**b**), sediment-spring (**c**), and sediment-autumn samples (**d**).


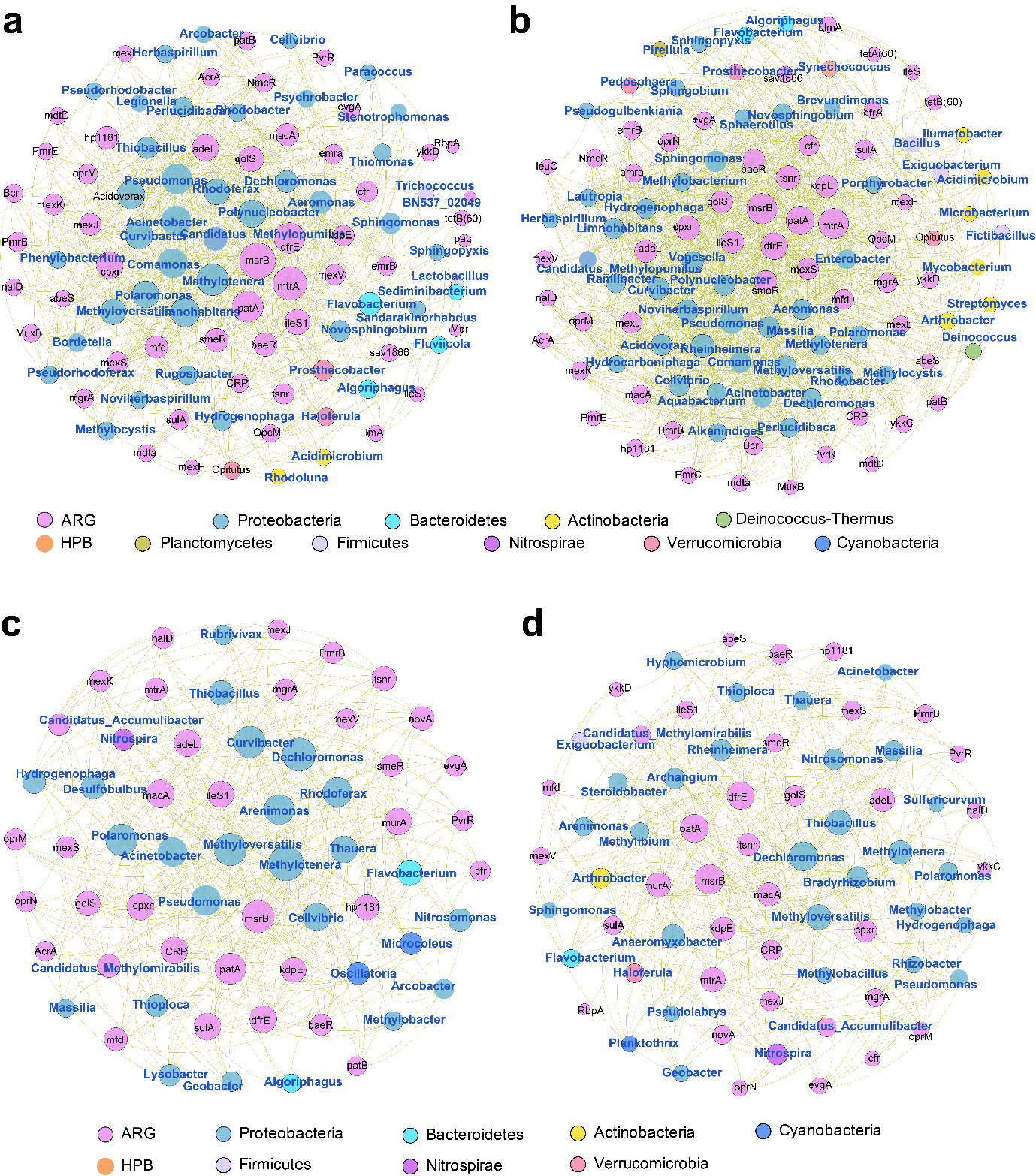


**Figure S9.** Networks displaying ARG hosts carrying multiple ARGs in water-spring (**a**), water-autumn (**b**), sediment-spring (**c**), and sediment-autumn samples (**d**) of the Yangtze River. The sizes of nodes correspond to the connection degree.


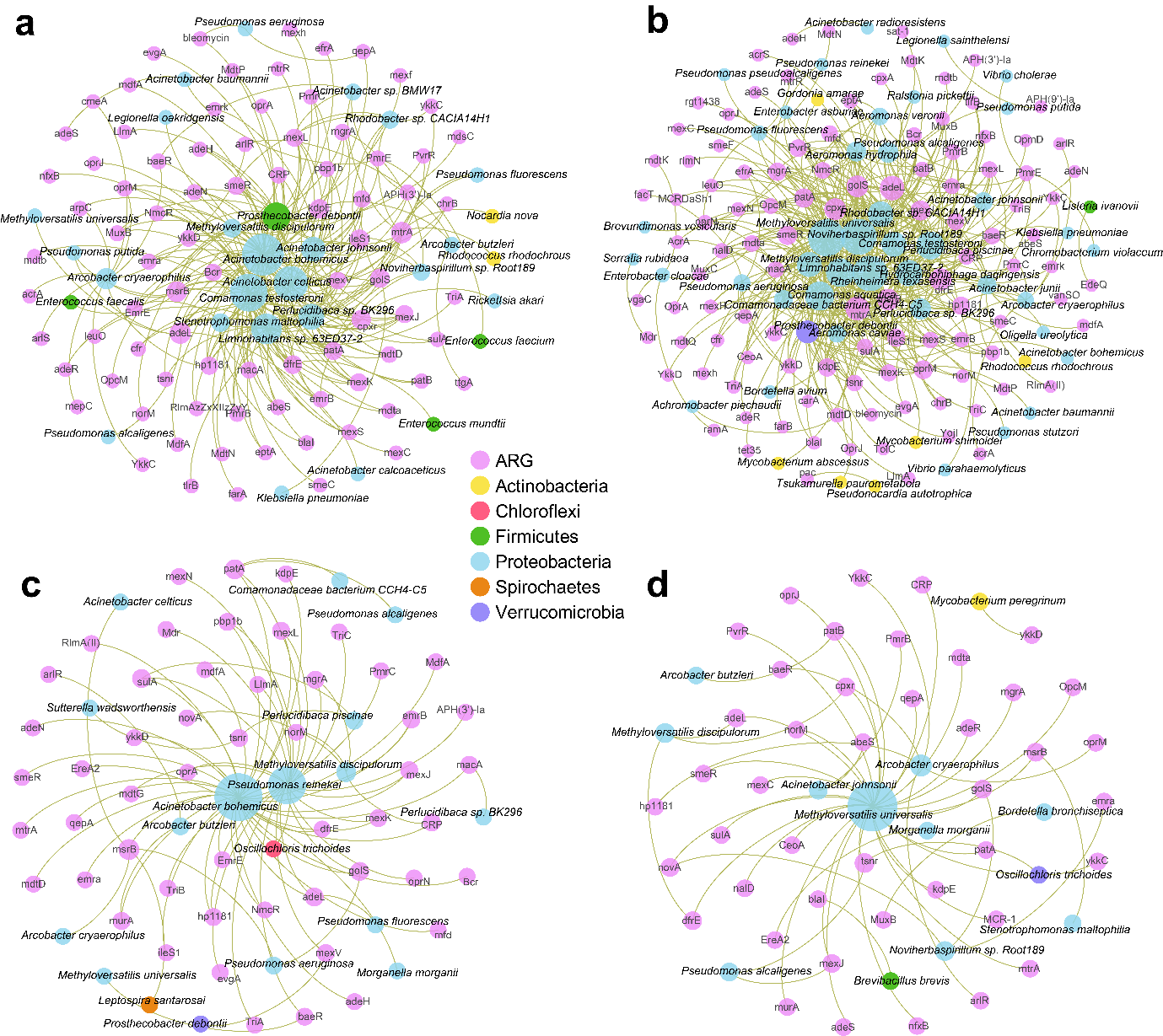


**Figure S10.** Networks displaying HPB carrying multiple ARGs in water-spring (**a**), water-autumn (**b**), sediment-spring (**c**), and sediment-autumn samples (**d**) of the Yangtze River. The sizes of nodes correspond to the connection degree.


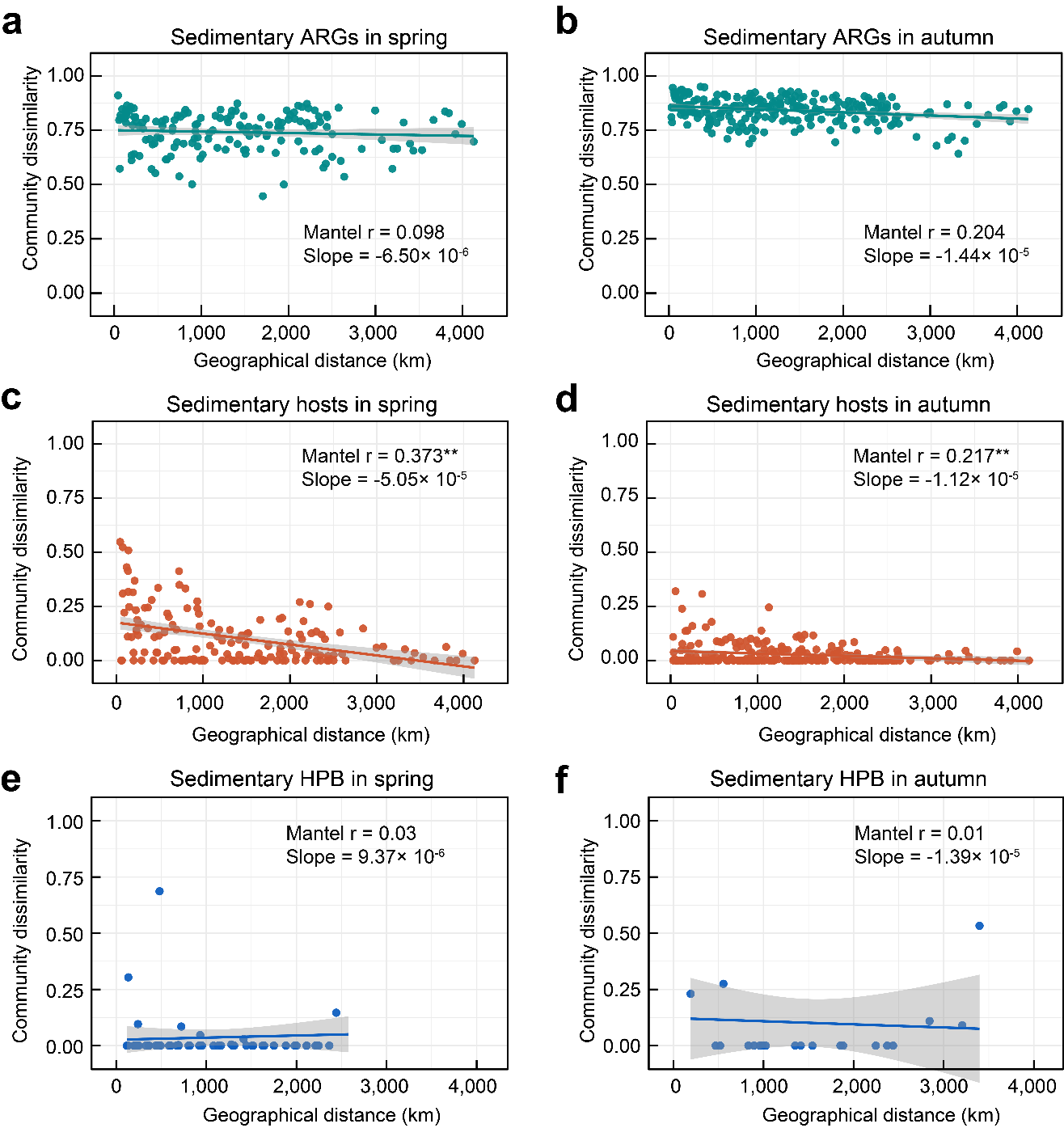


**Figure S11.** Distance-decay relationships of Bray-Curtis similarity of sedimentary ARGs in spring (**a**) and autumn (**b**), hosts in spring (**c**) and autumn (**d**), and HPB with the geographical distance in spring (**e**) and autumn (**f**). Mantel-Spearman correlations (r) and probabilities (significance codes: ***≤ 0.001 **≤ 0.01 *≤ 0.05) are provided. Solid lines indicate the ordinary least squares linear regression across all samples. Slopes of regression lines are also provided.


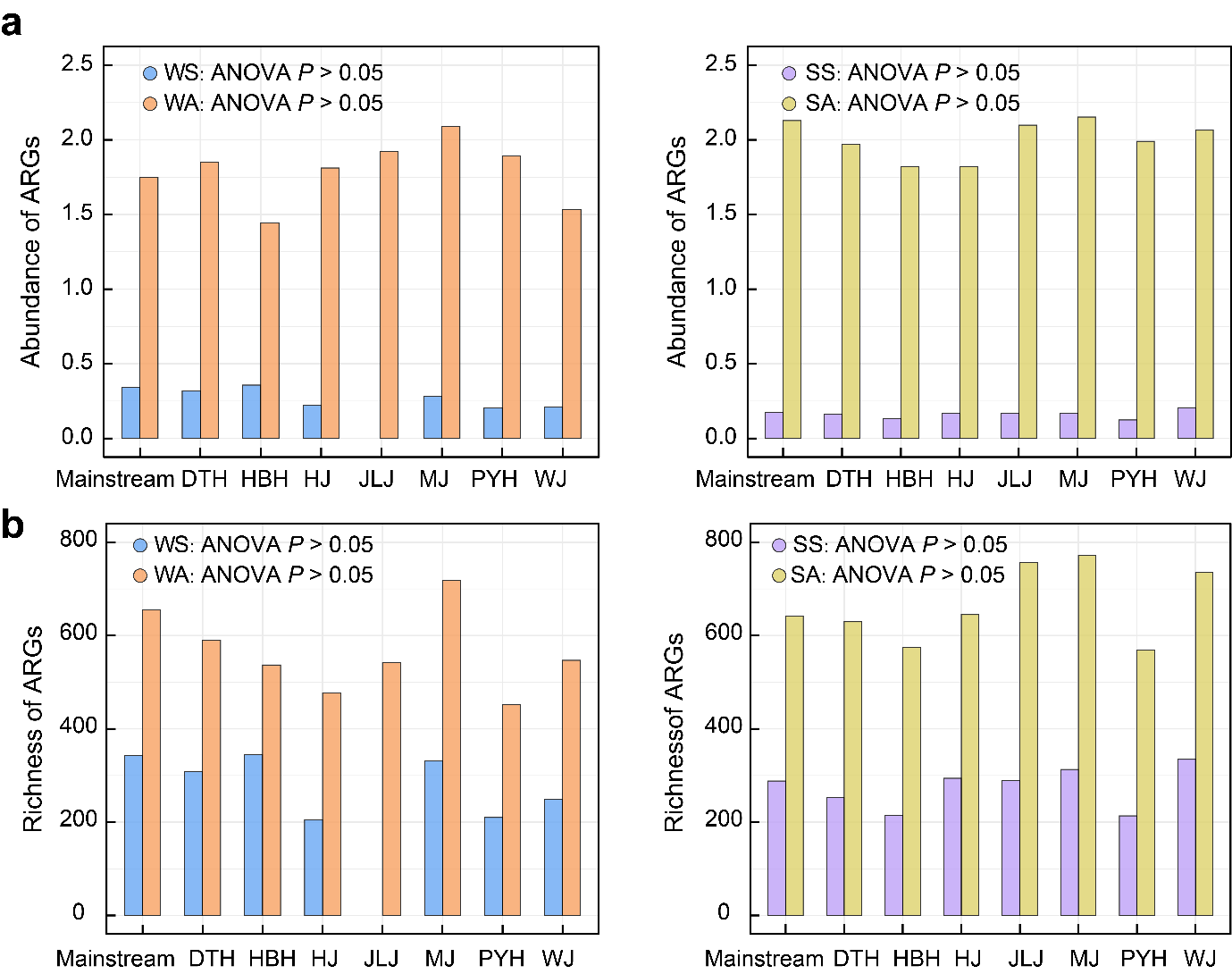


**Figure S12**. (**a**) Abundance (copy of ARG per copy of 16S-rRNA gene) and (**b**) richness of planktonic and sedimentary ARG subtypes in mainstream and seven tributaries (DTH: Dongtinghu, HBH: Huangbohe, HJ: Hanjiang, JLJ: Jialingjiang, MJ: Minjiang, PYH: Poyanghu, and WJ: Wujiang) along the Yangtze River. WS, WA, SS, and SA refer to water-spring, water-autumn, sediment-spring, and sediment-autumn samples, respectively.


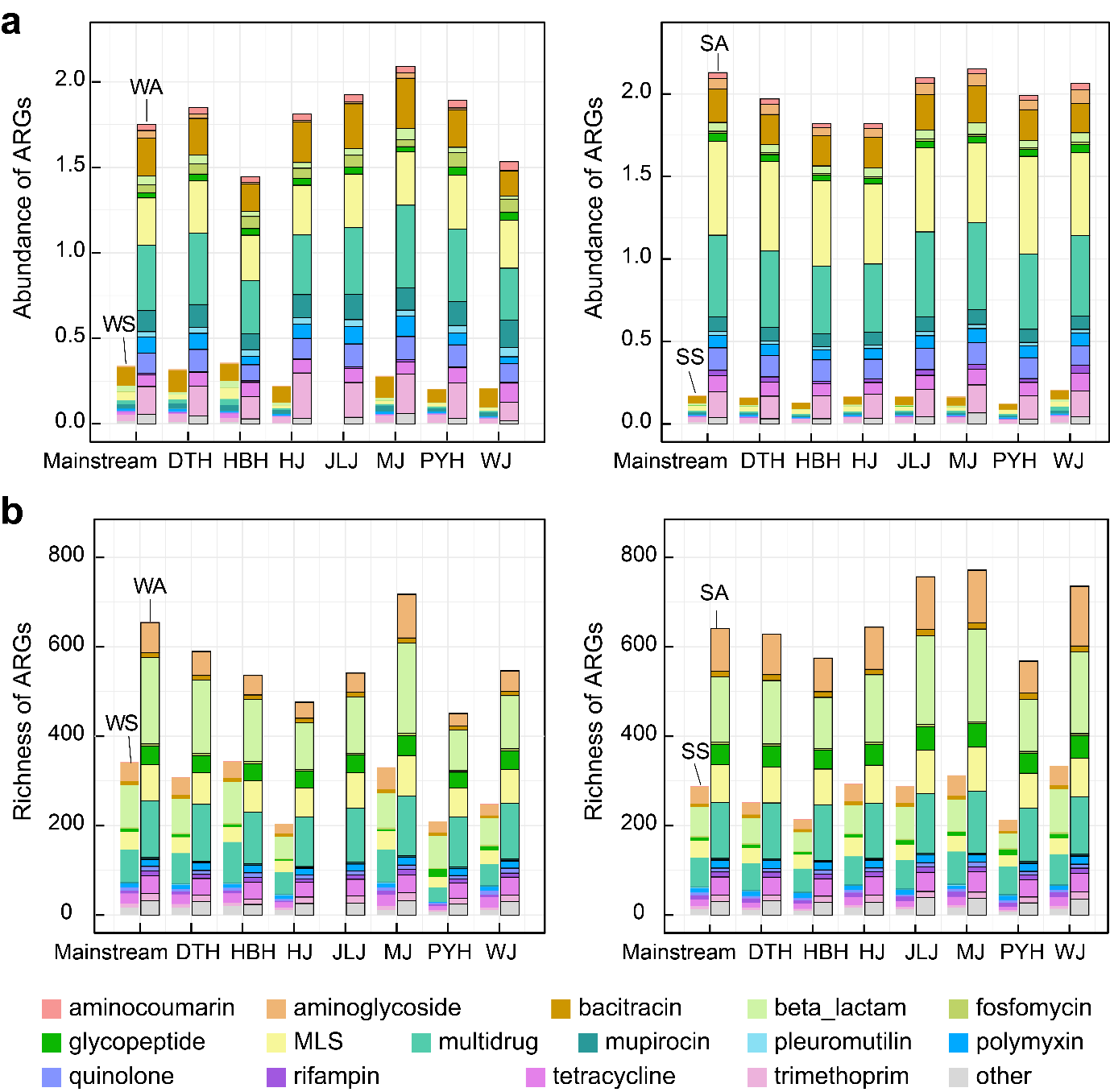


**Figure S13**. (**a**) Abundance (copy of ARG per copy of 16S-rRNA gene) and (**b**) richness of planktonic and sedimentary ARG types in mainstream and seven tributaries (DTH: Dongtinghu, HBH: Huangbohe, HJ: Hanjiang, JLJ: Jialingjiang, MJ: Minjiang, PYH: Poyanghu, and WJ: Wujiang) along the Yangtze River. WS, WA, SS, and SA refer to water-spring, water-autumn, sediment-spring, and sediment-autumn samples, respectively.


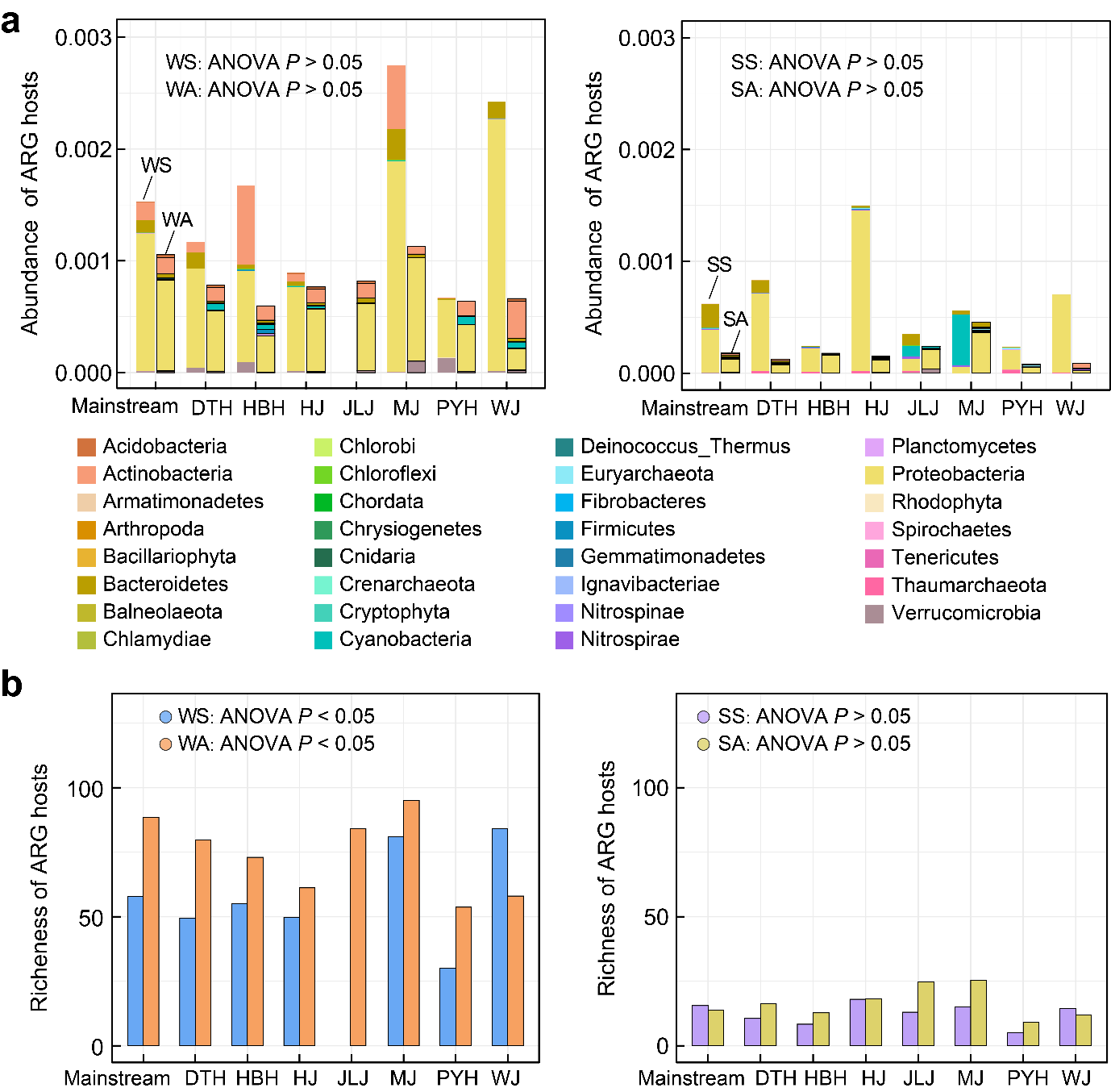


**Figure S14**. (**a**) Abundance (coverage/Gb) and (**b**) richness of planktonic and sedimentary ARG hosts in mainstream and seven tributaries (DTH: Dongtinghu, HBH: Huangbohe, HJ: Hanjiang, JLJ: Jialingjiang, MJ: Minjiang, PYH: Poyanghu, and WJ: Wujiang) along the Yangtze River. WS, WA, SS, and SA refer to water-spring, water-autumn, sediment-spring, and sediment-autumn samples, respectively.


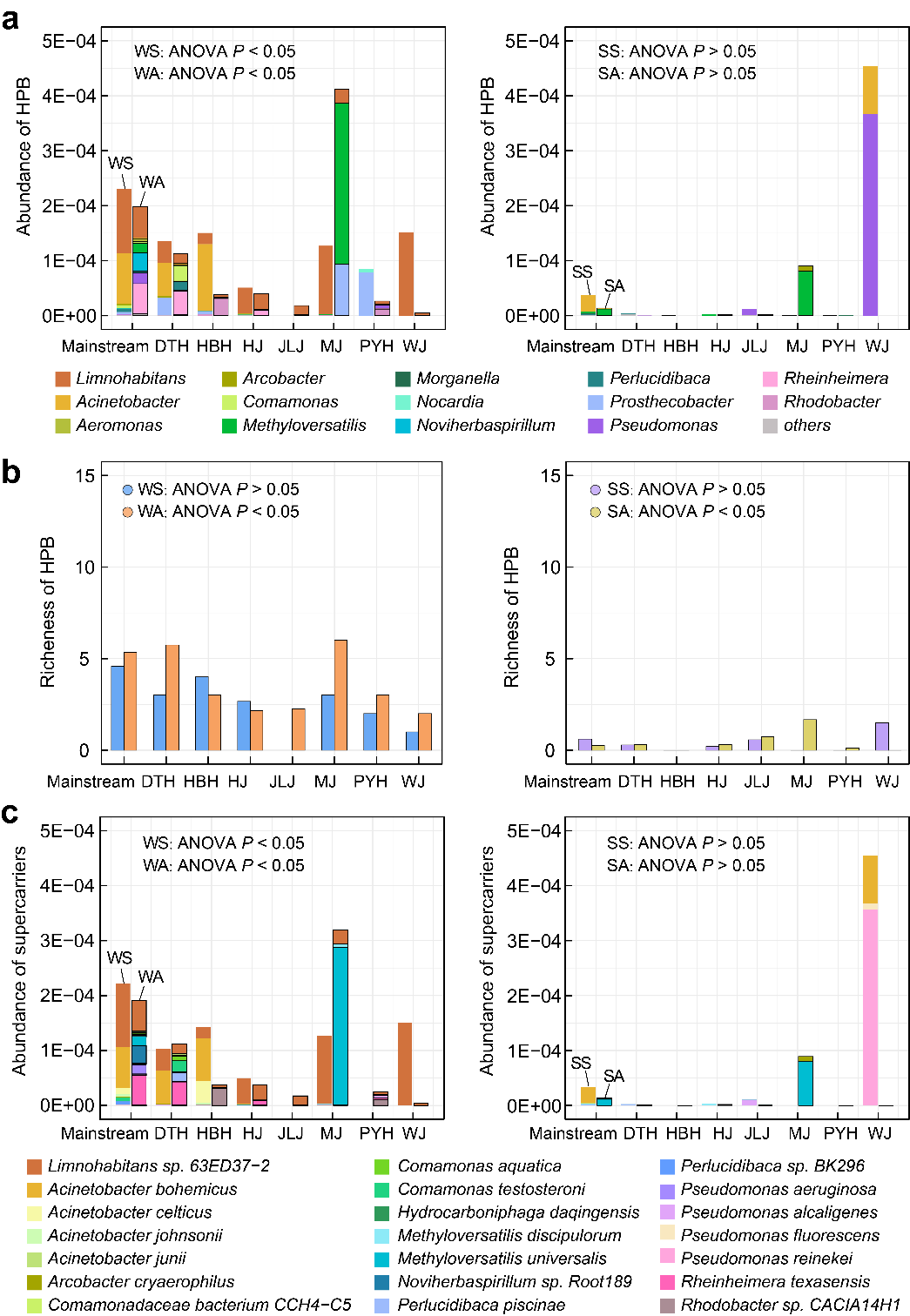


**Figure S15.** (**a**) Abundance (coverage/Gb) and (**b**) richness of planktonic and sedimentary HPB in mainstream and seven tributaries (DTH: Dongtinghu, HBH: Huangbohe, HJ: Hanjiang, JLJ: Jialingjiang, MJ: Minjiang, PYH: Poyanghu, and WJ: Wujiang) along the Yangtze River. (**c**) Abundance (coverage/Gb) of planktonic and sedimentary supercarriers in mainstream and seven tributaries. WS, WA, SS, and SA refer to water-spring, water-autumn, sediment-spring, and sediment-autumn samples, respectively.


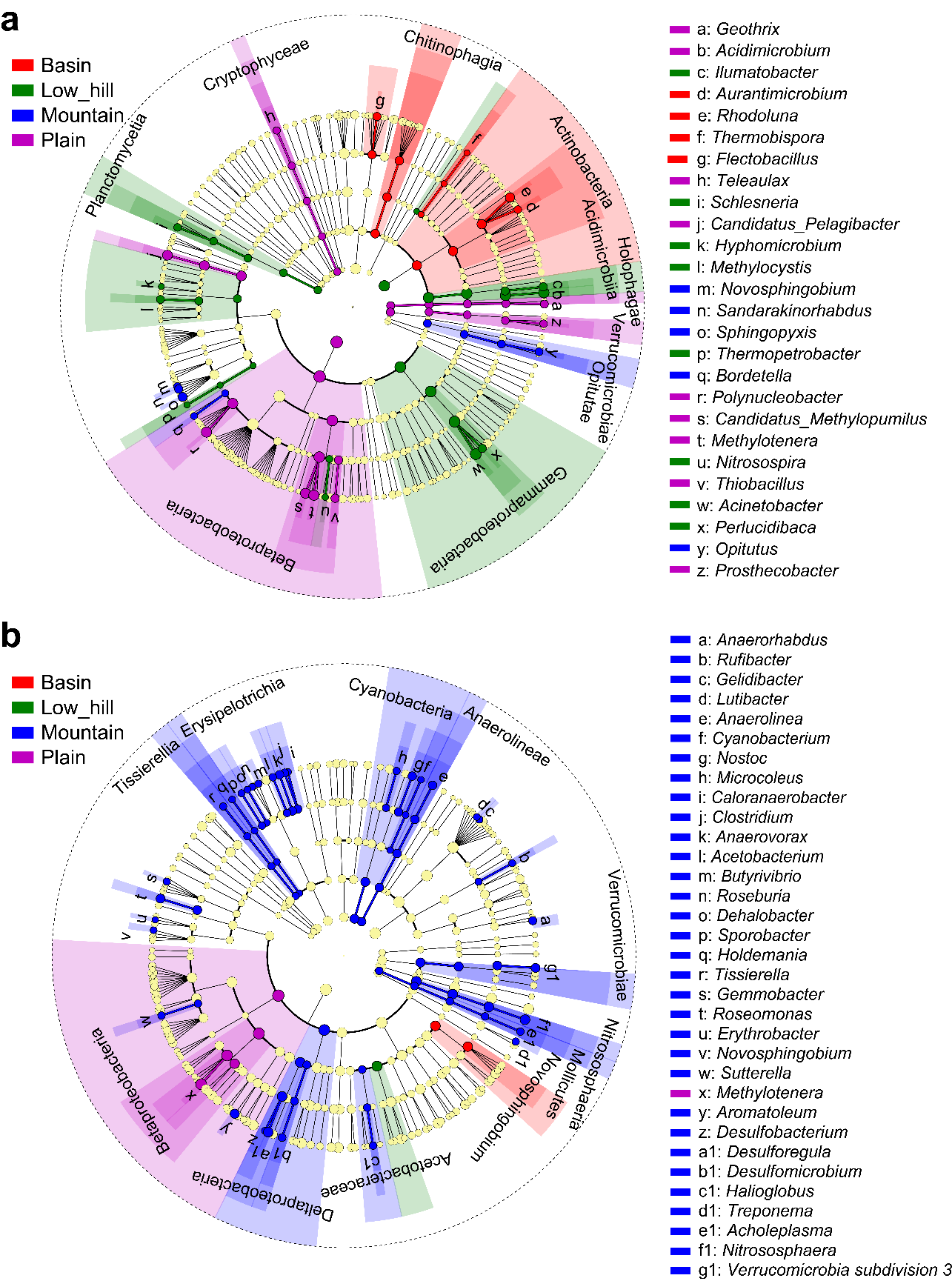


**Figure S16.** LEfSe cladogram depicting the taxonomic differences of ARG hosts in water (**a**) and sediment (**b**) in spring for four landform types. Differentially abundant taxa (biomarkers) are colored according to their most abundant landform habitats.


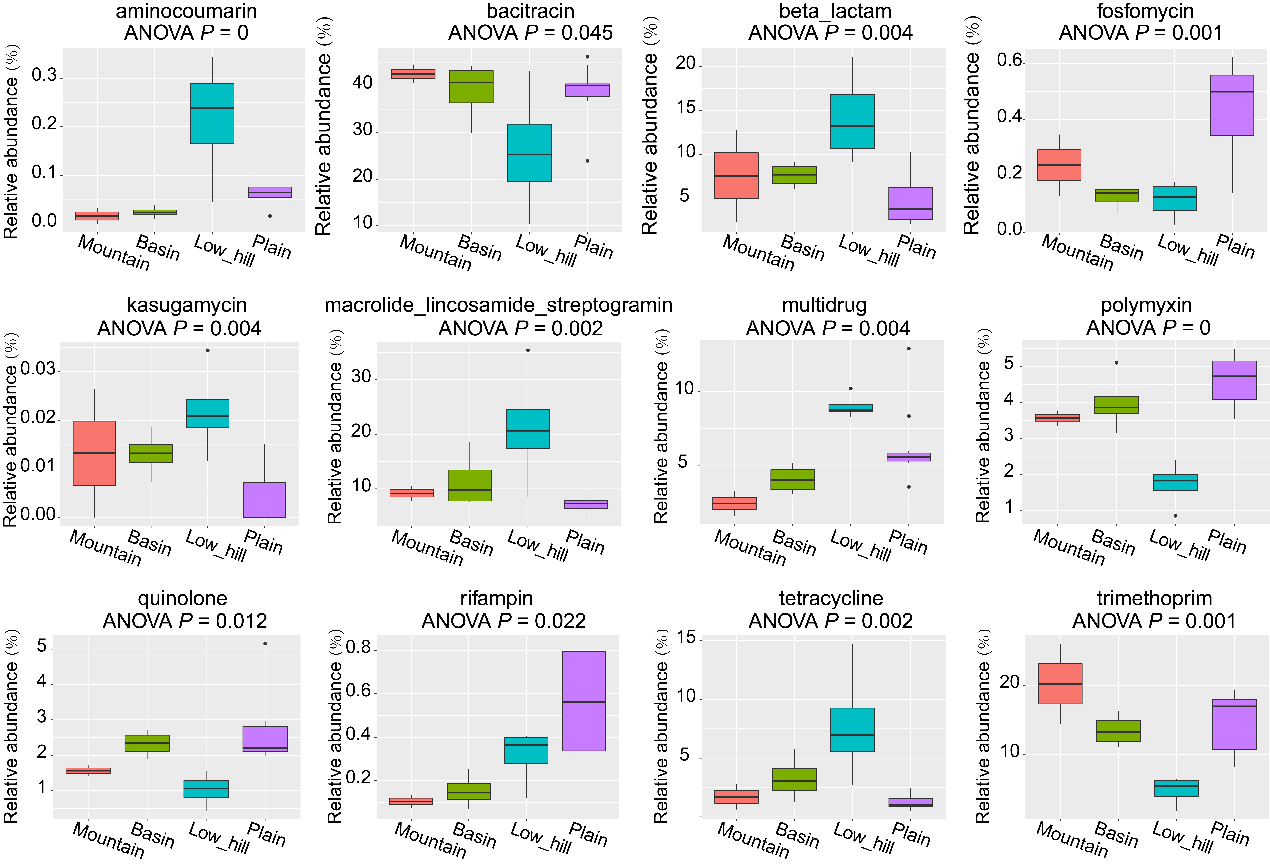


**Figure S17.** ARG types (water-spring group) exhibiting significant differences among four landform types along the Yangtze River.


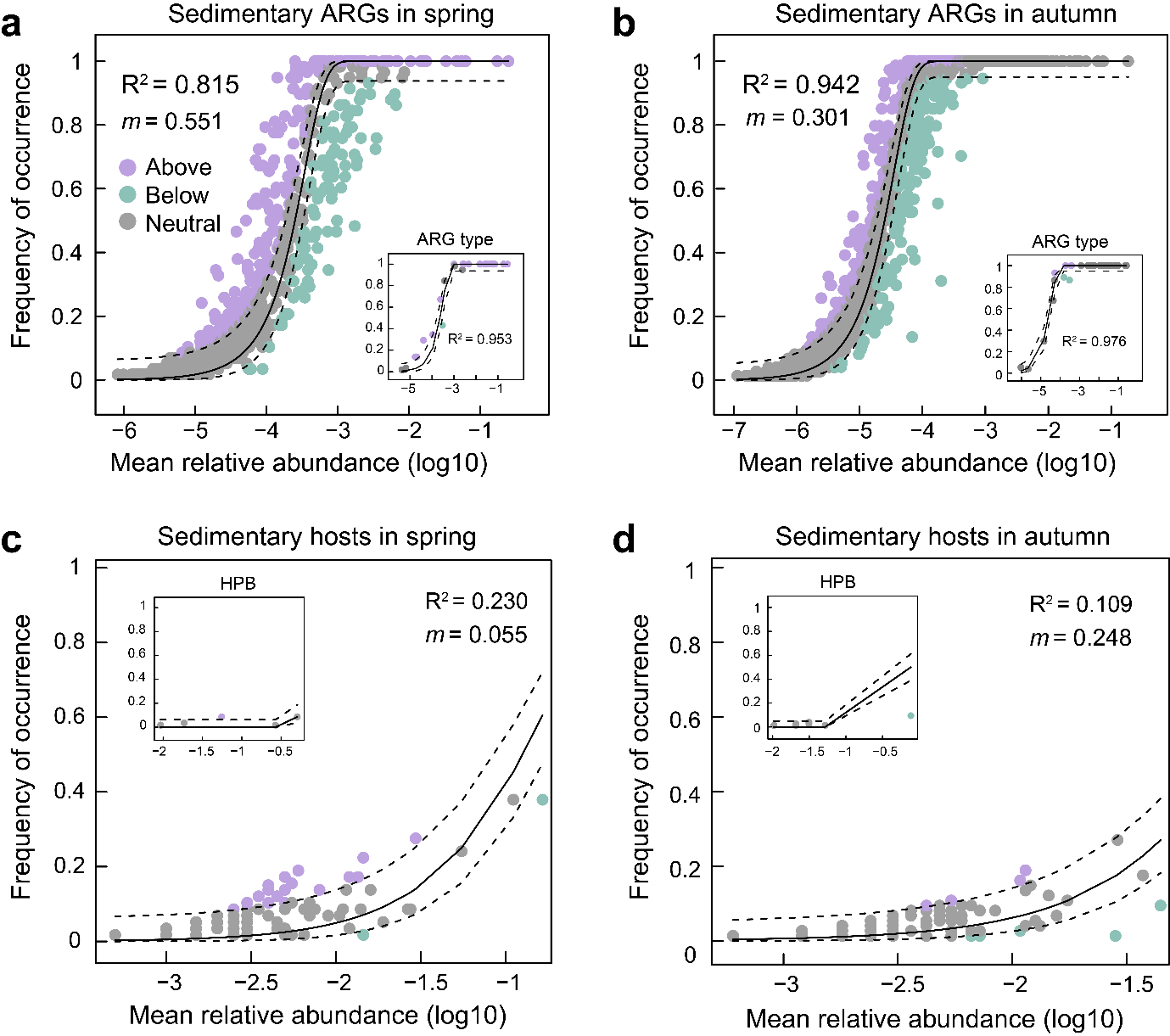


**Figure S18.** Occurrence frequency of sedimentary ARG subtypes in spring (**a**) and autumn (**b**) as well as hosts in spring (**c**) and autumn (**d**) fitted to mean relative abundance using Sloan *et al*.’s neutral community model. Inserts in (**a**-**b**) and (**c**-**d**) show the neutral community model fits to ARG type and HPB. Purple and green dots indicate ARGs/hosts that occur more (‘Above’) and less (‘Below’) frequently than given by the neutral model (gray dots, ‘Neutral’). R^2^ indicates the fit to the neutral community model, and *m* indicates the immigration rate. Dashed lines represent 95% confidence intervals about the model prediction.


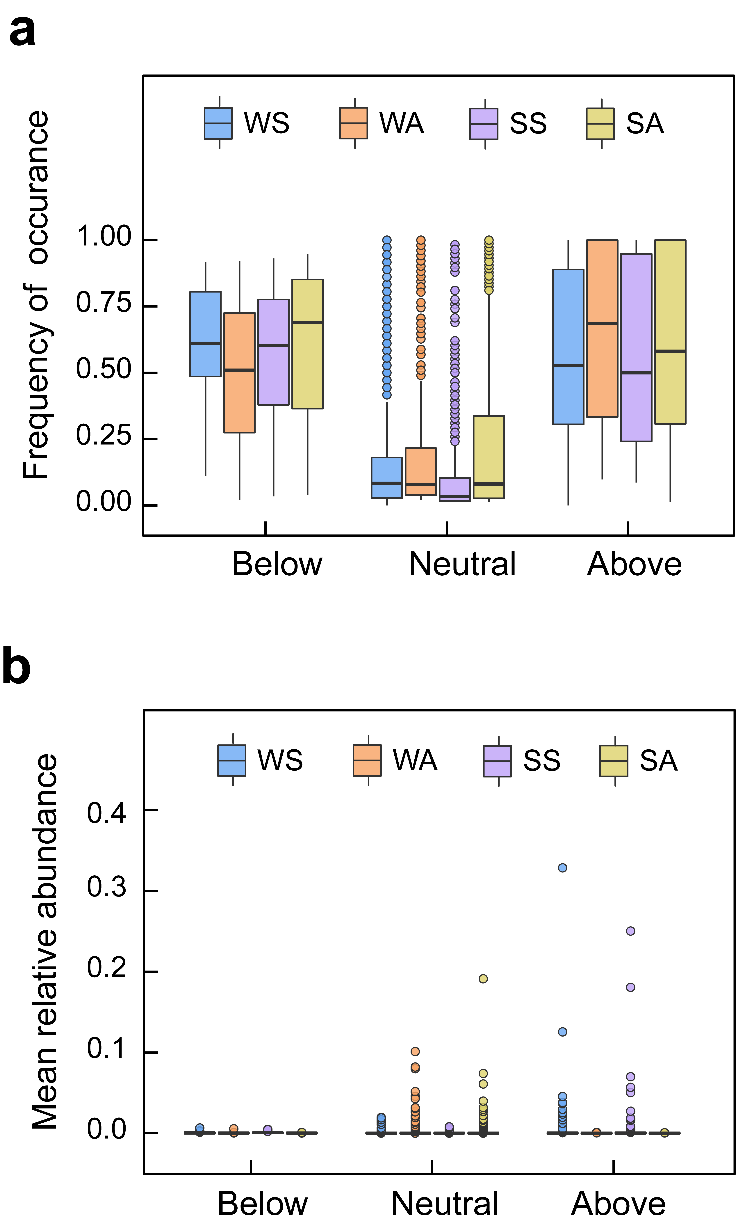


**Figure S19.** Distribution of occurrence frequency (**a**) and mean relative abundance (**b**) of ARGs in different sampling groups. ‘Above’ and ‘Below’ indicate ARGs that occur more and less frequently than given by the neutral model (‘Neutral’).


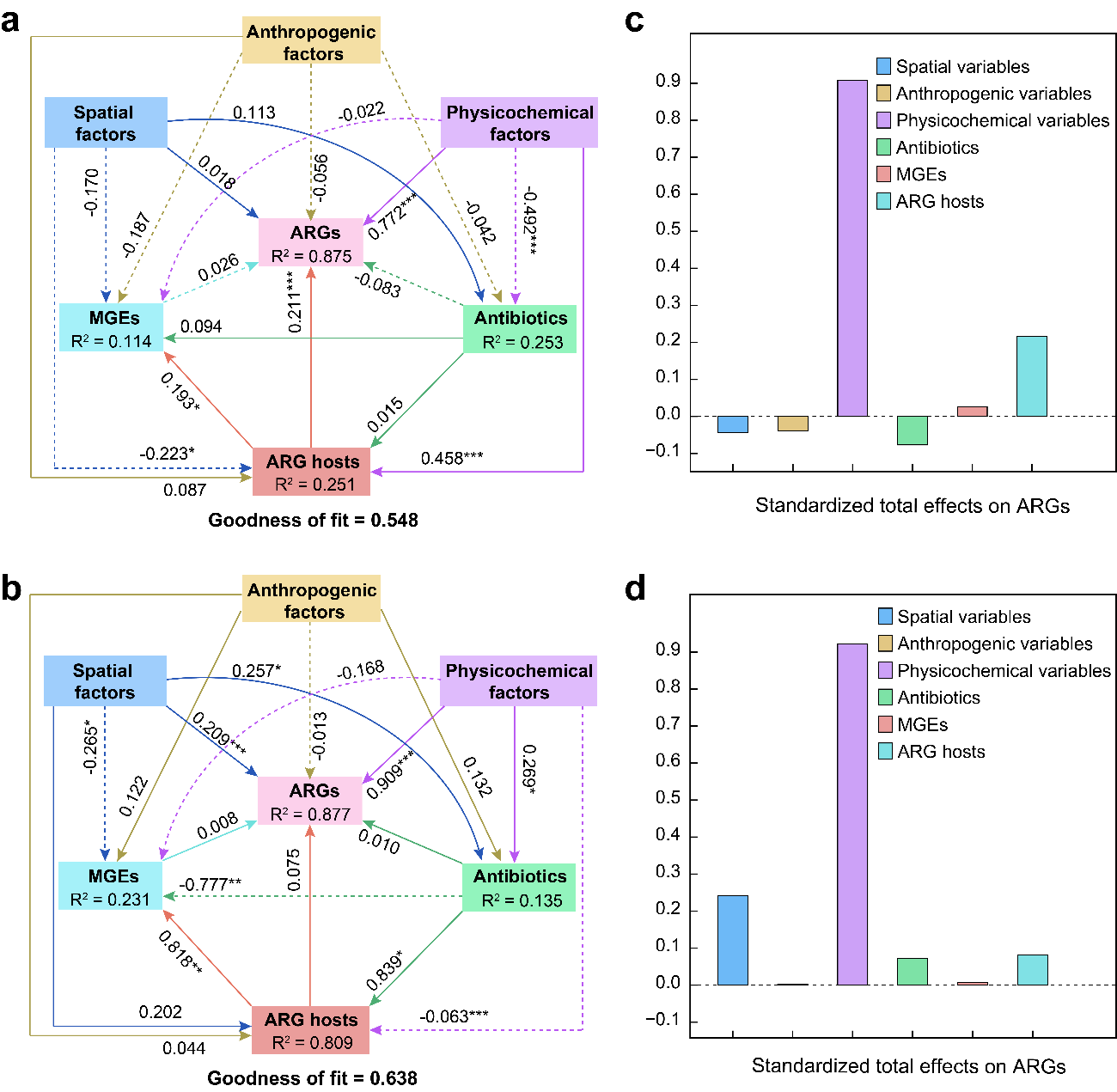


**Figure S20.** The partial least squares path models showing the effects of spatial variables, anthropogenic variables, physicochemical variables, antibiotics, MGEs, and ARG hosts on ARG compositions in water (**a**) and sediment (**b**) of the Yangtze River. Solid and dashed lines indicate positive and negative effects, respectively. Numbers adjacent to each arrow denote partial correlation coefficients (significance codes: ***≤ 0.001 **≤ 0.01 *≤ 0.05). R^2^ values display the proportion of variance explained for each factor. The bar-chart showing the standardized total effect of each factor on the ARG composition in water (**c**) and sediment (**d**).


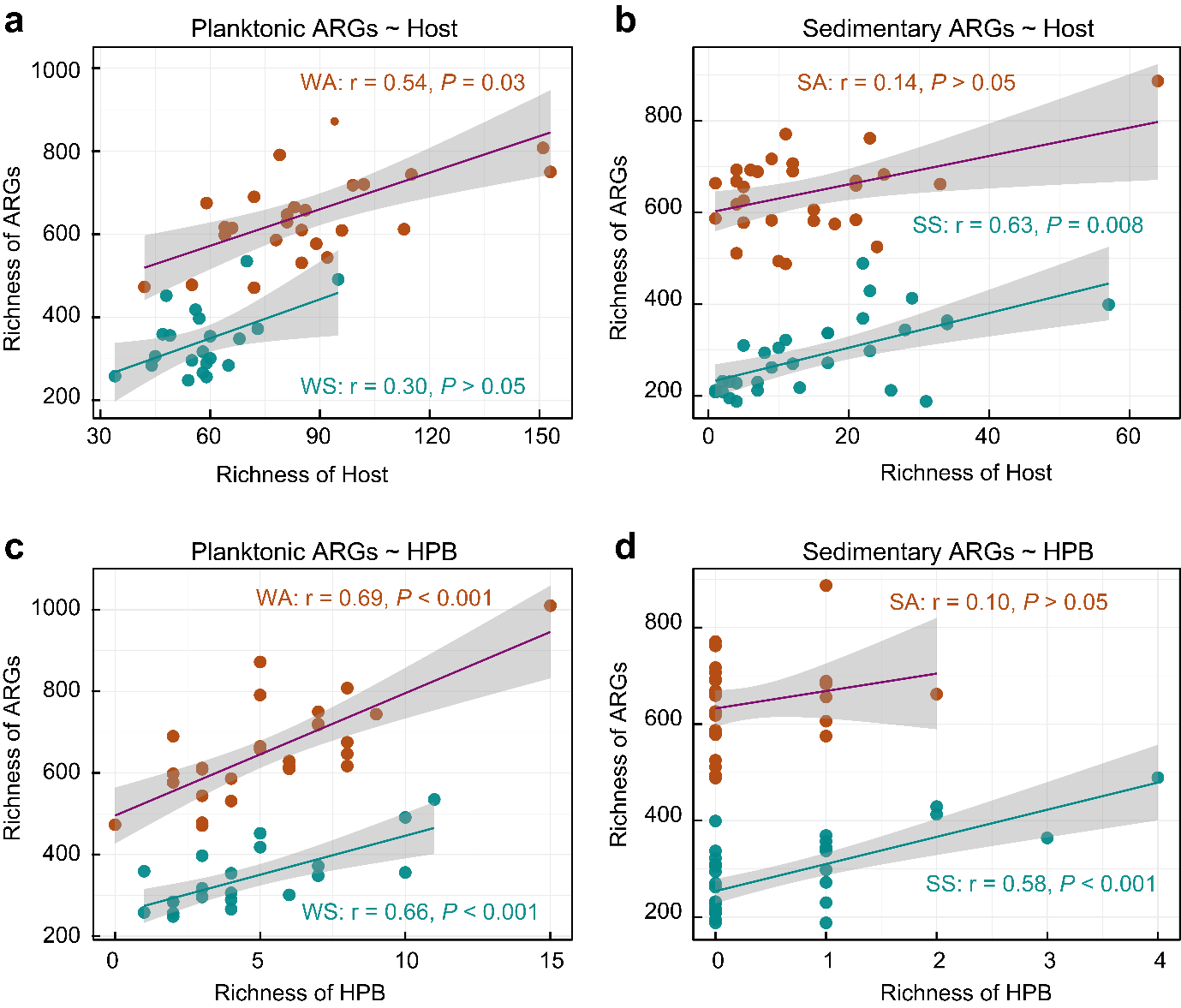


**Figure S21.** Spearman’s correlations (R) between the richness of ARG host and ARGs in water (**a**) and sediment (**b**), and between the richness of HPB and the richness of ARGs in water (**c**) and sediment (**d**). Ordinary least square linear regressions and 95% confidence intervals are also displayed.
